# Federated learning for fair autism spectrum disorder screening across age-heterogeneous populations

**DOI:** 10.3389/fdgth.2026.1760849

**Published:** 2026-04-07

**Authors:** Siwar Rekik, Sajid Mehmood, Lamia Berriche

**Affiliations:** 1 Computer Science Department, College of Computer and Information Sciences, Prince Sultan University, Riyadh, Saudi Arabia; 2 Department of Computer Science, University of Engineering and Technology, Taxila, Pakistan

**Keywords:** autism spectrum disorder, federated learning, fairness, healthcare artificial intelligence, machine learning, multi-institutional collaboration, personalized machine learning, privacy preservation

## Abstract

**Introduction:**

The detection of Autism Spectrum Disorder (ASD) remains challenging due to the heterogeneity of behavioural manifestations, limited dataset availability, and strict privacy requirements. Conventional centralized machine learning approaches often suffer from overfitting and limited generalizability across different age groups. This study proposes a federated learning (FL) framework to enable collaborative ASD screening across children, adolescents, and adults without sharing sensitive patient data.

**Methods:**

A federated learning framework was implemented and benchmarked using multiple FL algorithms, including FedPer, pFedMe, and q-FedAvg. These were compared with traditional centralized machine learning models such as Support Vector Machine (SVM), Random Forest, K-Nearest Neighbors (KNN), and J48. Data preprocessing involved imputation, encoding, scaling, feature selection, and Synthetic Minority Over-sampling Technique (SMOTE) to address missing values, categorical variables, and class imbalance. Model performance, fairness, robustness under non-IID conditions, computational efficiency, and communication costs were evaluated.

**Results:**

Customized federated learning approaches achieved superior global accuracy of 97.2% for children, 89.5% for adolescents, and 86.8% for adults. The proposed framework demonstrated improved fairness and robustness in heterogeneous non-IID environments compared to centralized models, while maintaining computational and communication efficiency.

**Discussion:**

The findings indicate that personalized federated learning provides a scalable, accurate, and privacy-preserving solution for ASD screening across diverse age groups. By bridging advanced machine learning techniques with ethical clinical practice, the proposed framework supports responsible and effective ASD detection in real-world healthcare settings.

## 1 Introduction

Autism Spectrum Disorder (ASD) is an ambiguous neurodevelopment disorder characterized by a continued inability to communicate, socialize, and exhibit repetitive or limited behavior patterns (1–3). ASD has been growing steadily over the last two decades, with recent reports estimating that the prevalence of ASD is about 1 in 100 children globally (4). Early and accurate risk assessment is essential because early interventions have been reported to positively influence development, academic and quality of life (5). Nevertheless, existing practice of diagnosing ASD is based on long-term behavior assessment procedures by the expert community that are resource-consuming and subject to subjectivity and access unequal opportunities between regions (6).

Machine learning (ML) has become an innovative mechanism due to the potential to aid the screening of ASD to detect discriminative patterns of behavioural and demographic data (7, 8). A number of studies have shown that traditional supervised learning algorithms, including Support Vector Machines (SVM), Random Forests (RF), and Decision Trees), can be used to classify ASD traits with a high level of accuracy (9–11). Although the results are encouraging, the majority of the current methods are trained on a single dataset and thus limit their generalizability and make overfitting a plausible issue to consider (47). Furthermore, there is a lack of and fragmentation of data, which is also a major challenge, with clinical and behavioural records being spread throughout institutions and being subject to heavy regulations of privacy (12, 13).

To overcome these shortcomings, the recently proposed federated learning (FL) is a collaborative training paradigm that allows several data owners to train a common model without storing sensitive records in a central place (14, 15). FL also helps save patient privacy by merging model parameters rather than raw data, and enhances the resistance to heterogeneous data sources (16). Despite the fact that FL has been investigated in more general healthcare fields like medical imaging and electronic health records, it has not been fully investigated in ASD screening before (17).

This paper provides a comparative architecture, where we compare traditional ML classifiers and federated learning algorithm in the context of predicting ASD. Based on several publicly available datasets on ASD of children, adolescents and adults, we explore trade-offs between centralised models and Privacy-Aware distributed training. We may make the following contributions:

- Perform extensive benchmarking of the four conventional supervised classifiers, i.e., SVM, Random Forest, KNN, and J48, employing feature-selection methods to make robust centralised baselines on ASD prediction.
- Train ASD on Privacy-Aware federated learning framework based on collaborative learning over partitioned datasets without the exchange and sharing of the raw patient data.
- Carry out a comprehensive empirical analysis of centralized or federated models in child, adolescent and adult cohorts, with accuracy, precision, recall, F1-score and AUC being reported.
- Introduce novel understanding of generalisation, fairness and privacy by showing how federated learning can achieve greater robustness in non-IID training and can be used to scale and ethically responsibly screen ASD.

The purpose of this paper is to present the concept of Autism Spectrum Disorder (ASD) screening, instead of clinical risk assessment. Although machine learning models might discern patterns that correlate with high risk of ASD based on features that are derived through the use of questionnaires, it cannot provide clear risk assessment without the use of in-depth clinical assessment by the qualified professionals. Based on this, the suggested framework should facilitate large-scale screening and risk assessment and has no intention to substitute and automate clinical screening decision-making. This difference is preserved throughout the manuscript in order not to overestimate clinical implications.

The other parts of this paper are structured in the following manner. Part II conducts a literature review on related work on ML and federated learning in ASD and healthcare. Section 3 presents the datasets, preprocessing procedures and classification models. In Section 4, the results are presented and discussed. Lastly, the paper ends with a Discussion in Section 5 and Conlusion in Section 6, which gives thenfuture research directions.

## 2 Related work

In recent years, research on automated detection of Autism Spectrum Disorder has seen a rapid growth, accompanied by new technological breakthroughs in classical machine learning, deep learning, questionnaire-based sensing, and, recently, federated learning (18). Classical that are founded on structured behavioural questionnaires and engineered features still provide a competitive performance; large-cohort and AutoML experiments have demonstrated that small and clinically-informed feature sets and engineered pipelines can be highly accurate and interpretable on child-related datasets (19). These centralised models illustrate the possibilities of machine learning in the ASD screening process, though it assumes the possibility to merge sensitive patient information in a single repository, which is not a feasible assumption, considering the modern privacy laws and institutional governance limitations. Representative centralized work and systematic review report high within-dataset performance, however also highlight the danger that this type of model will not work when utilized in other institutions or age groups due to distributional heterogeneity and privacy limitations (20).

Representational capacity has also been extended through deep learning and questionnaire-based research through integrating facial video, EEG, eye-tracking, and neuroimaging (21). Recent papers have seen significant accuracy gains with CNN, CNN-LSTM and hybrid models on modality-rich models and other papers have combined explainability techniques with models to enhance clinical trust. However, these deep architectures are typically trained in a centralised environment and thus they do not test cross-site privacy and are not systematically tested on their robustness to heterogeneous client populations, or to differences in development-stage. There is thus no empirical evidence in the literature demonstrating whether high-capacity, questionnaire-based deep models maintain their performance and interpretability when trained in a distributed, Privacy-Aware manner to be used by various clinics (22).

FL has become a principled solution to the obstacles to privacy and governance and has proven to be successful in various medical fields (23). The number of works on FL-enabled pipelines has been increasing since 2023 in the ASD field. Initial federated experiments showed that with classical ML classifiers modified to local training and server-side aggregation, it was feasible, and subsidiary experiments generalised FedAvg-style aggregation approach to CNN and CNN-LSTM models of behavioural and facial modalities, where in some cases almost centralised accuracy was demonstrated (24). Other more recent publications suggested federated workflows which are procedural mixes of local selection of features and global model aggregation to provide greater levels of transparency as well as maintain data locality. These federated ASD studies demonstrate that it is possible to train good models without centralising raw data but also indicate two regular limitations: most experiments only test a limited number of FL algorithms (usually FedAvg), and they do not often test personalization or fairness (25).

One methodological gap in the current federated ASD literature is the consideration of non-IID heterogeneity among the clients (26). There is a systematic difference between clinical sites in terms of age (children, adolescents, adults), modality (behavioural questionnaires, video, EEG, fMRI), class imbalance, local labelling. Even though personalization (e.g., FedBN, FedPer, FedProx, pFedMe) and fairness-conscious aggregators (e.g., q-FFL, FairFed) have been implemented in the general FL literature to address client drift and limit per-client disparities, they have not undergone extensive benchmarking on ASD datasets. As a result, it is not clear how standard FL, personalised FL and fairness-aware FL compare based on cohorts of age-differentiated and questionnaire-based inputs in terms of accuracy, per-client robustness and equitable utility (27).

The other gap is disintegration of assessment systems (28). In the context of centralised ML, deep learning, and FL approaches, previous research usually compares the methods independently, utilising various datasets, cohort splits, preprocessing options, and evaluation measures. Such fragmentation does not allow making rigorous cross-method conclusions and reduces arguments on the applicability of FL in clinical screening of ASD. The divergent nature of benchmarking pipelines with classical centralized benchmarks, FedAvg, several personalized FL schemes, and fairness-conscious aggregators even across age-diverse datasets does not allow the community to learn trade-offs between performance, privacy, and fairness (29).

The current study fills these gaps by offering a multi-method benchmark on a systematic comparison of both classical machine learning baselines, centralized deep models, FedAvg, various personalization schemes, including FedBN, FedPer, FedProx, pFedMe, and fairness-sensitive aggregation, which includes q-FedAvg, on three public ASD cohorts of children, adolescents, and adults. The assessment criterion is on a global measure (accuracy, precision, recall, F1, AUC) and client-specific measures of fairness, assuming non-IID partitioning. Client-specific fairness measures include per-client accuracy, sensitivity, specificity, and AUC evaluated independently on each client’s test set, as well as standard deviations between clients, worst-case accuracy. Furthermore, we compared the local preprocessing and communication-overheads for the different federated techniques. We can understand the optimal balance of accuracy, generalization, privacy preservation, and fair client utility of the methods employed in real-world federated ASD screening by harmonizing preprocessing, model selection and evaluations procedures across methods and cohorts and based on the results of the study. The suggested framework thus addresses the most significant gaps in the literature: (i) it assesses FL methods on age-heterogeneous, age-heterogeneous questionnaire-based ASD data; (ii) it compares many personalization and fairness strategies to centralized baselines; and (iii) it makes a reproducible evaluation execution pipeline, which can be used to make fair cross-method comparisons (20).

The following Table 1 provides an overview of the most up-to-date research and mentions its main modalities and the focus on methodology, as well as indicates the exact limitations that the present work addresses.

**Table 1:** Comparison of selected recent ASD and federated learning studies.

Study (selected)	Year	Methodology	Dataset/modality	Main strength	Key limitation (addressed here)
Lakhan et al. (20)	2023	Fed-enabled CNN–LSTM	questionnaire-based behavioural/visual (children)	High accuracy in child cohort; questionnaire-based FL pipeline	Single-age cohort; limited personalization and fairness evaluation
Mohammad- ifar et al. (25)	2023	Federated SVM (SVCFL)	Questionnaire-based child datasets	Lightweight FL with classical ML; feasibility demonstrated	Narrow algorithmic scope; no fairness or personalization
Gupta et al. (22)	2024	Quantized 1D CNN + FL	ABIDE-1 (fMRI)	Resource-efficient FL on neuroimaging; strong performance	Single modality; limited cross-cohort evaluation
Alshammari et al. (30)	2024	Explainable FL + deep models	Toddler EEG/behavioural datasets	Combines XAI with FL for transparency	Early-stage; lacks personalization benchmarking
Mohammad Syfullah et al. (31)	2024	Federated ML pipeline	Mixed child/adolescent datasets	Shows broader FL feasibility	Early preprint; heterogeneity and fairness unexplored
Kranthi et al. (32)	2025	Federated CNN–LSTM	Behavioural video/time-series	Spatiotemporal modeling in FL	Limited evaluation across developmental/age stages
Guan et al. (33)	2024	FL for medical imaging	Multi-hospital imaging datasets	Demonstrates FL feasibility in clinical imaging settings	Not ASD-specific; does not address behavioural/tabular FL
Daliri et al. (27)	2025	Fair and personalized FL methods	Benchmark FL datasets	Addresses fairness and client heterogeneity theoretically	Not applied to ASD or clinical screening datasets
Setu et al. (29)	2025	Reviews/surveys	Multiple ASD modalities	Synthesis of trends and methodological needs	Lack empirical FL or fairness benchmarks for ASD
Almadhor et al. (34)	2025	Secure questionnaire-based FL + XAI	Eye-tracking, kinematics, EEG	Privacy + explainability integration proposals	Limited comparative experiments; small-scale evaluations

The above synthesis indicates that although FL has already become a viable and promising direction of ASD screening, there is no literature to date that offers a single, reproducible benchmarking study which unites centralised baselines, FedAvg, many different variants of personalization, and fairness-conscious aggregation across age-heterogenous cohorts with questionnaire-based inputs. The positioned gap is precisely bridged by the current work which has brought together preprocessing, model architectures, federated algorithms, and evaluation metrics and reported both global and per-client fairness results. This integrated assessment can explain what methods can be strong in clinical heterogeneity and which need additional methodological developments to be used fairly.

## 3 Methodology

**Figure 1:**
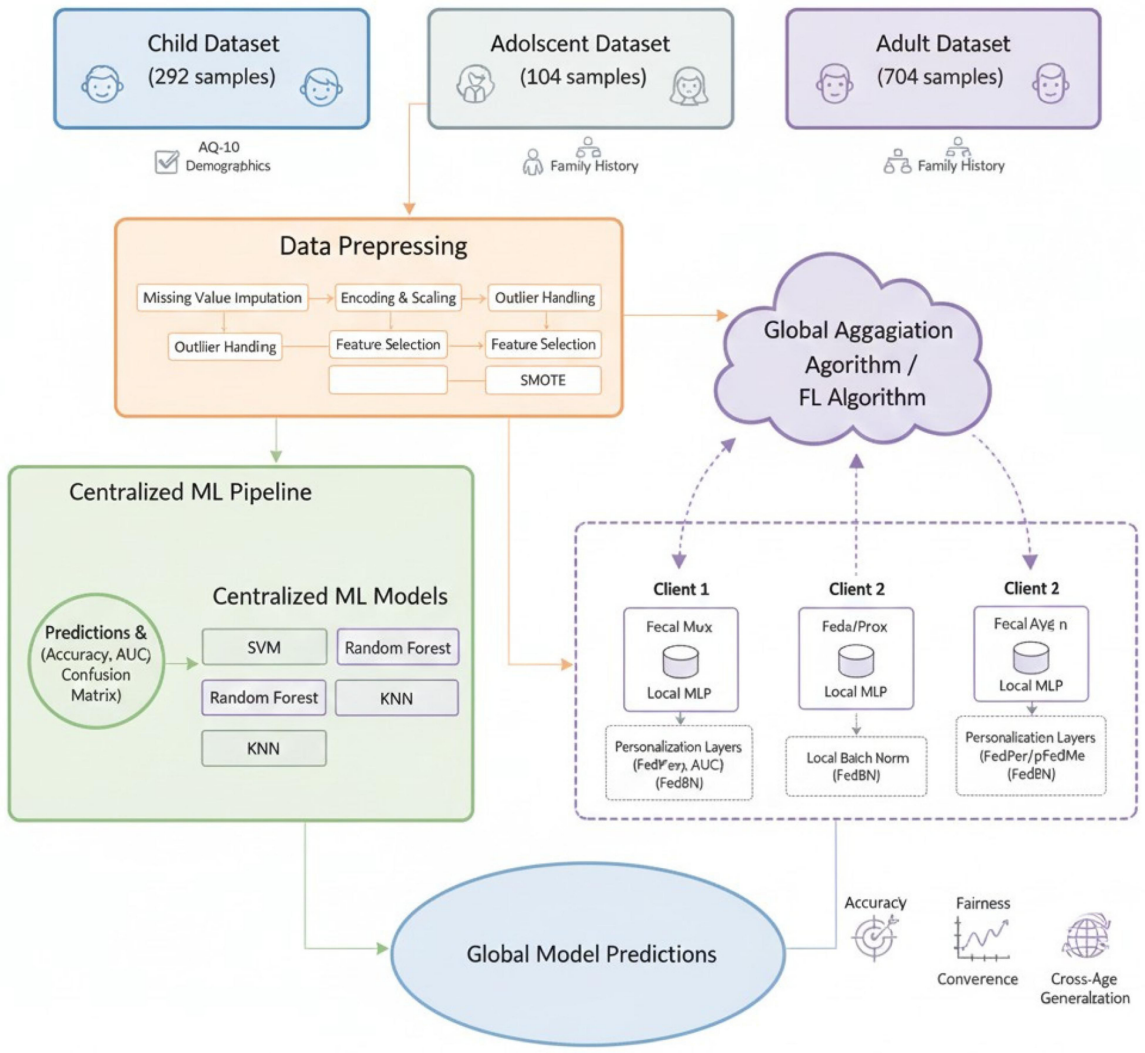
ASD model architecture.

The Methodology section will provide the description of the datasets, preprocessing pipeline, machine learning baselines, and federated learning framework used in the creation of the Privacy-Aware ASD screening system using heterogeneous age cohorts. It outlines the properties of the child, adolescent and adult cohorts; outlines the standard data preparation steps used prior to training the model; and outlines the centralised classifiers as well as the federated algorithms employed in this paper. In this part, one can also find the architectural description of the federated system shown in Figure 1, the setup of client nodes when the conditions are non-IID, and the general workflow that can promote collaborative learning without the necessity to share the raw data.

### 3.1 Datasets and non IID heterogeneity

The present research uses three publicly available Autism Spectrum Disorder (ASD) screening datasets, which represent the different developmental stages, including children, adolescents, and adults. Such datasets are leveraged in ASD machine-learning studies as they include structured behavioural questionnaires responses, demography and clinically validated ASD indicators. Their public availability ensures experimental reproducibility and supports ethical research. Above all, the ASD has a variant of different age ranges and thus the three distinct sets of data allow an analytic assessment of the generalisation of the centralised and federated models to a heterogeneous population.

The behavioural features of children aged 4-11 are found in the Child ASD Screening Dataset, which was first presented by Thabtah and colleagues and is located in the UCI Machine Learning Repository. It is also commonly used in the literature on ASD prediction because of its small size and validated AQ-10-based screening format to use in machine-learning benchmarks. The Adolescent ASD Dataset is a follow-up study on the same line of research and has a population segment of relatively smaller, yet significant population. Teenagers have variant behavioural patterns and different features of transition to social life, and the application of this data enables the model to gain age-specific differences. Adults have more heterogeneous cognitive and social profiles. Combined, these datasets suggest a holistic and realistic reference point on the study of ASD screening in a variety of population groups and conforms to the real-world needs of federated learning.

Non-IID heterogeneity occurs when data distributions differ across federated clients (35, 36), manifesting in different ways in these datasets:

- *Feature Distribution Shift:* Input features vary across age groups, measured using Kolmogorov-Smirnov tests defined according to Equation 1
  $$D=\max_{x}|F_{1}(x)-F_{2}(x)|$$ (1)
  where $F_{1}(x)$ and $F_{2}(x)$ are the empirical cumulative distribution functions for a feature x for two different cohorts respectively.
- *Label Distribution Shift:* Which shows how ASD prevalence varies by developmental stage, measured using chi-square tests.
- *Sample Size Heterogeneity:* where cohorts contribute different numbers of participants.
- *Class Imbalance Heterogeneity:* where the proportion of ASD-positive cases varies between cohorts.

The main peculiarities of the datasets are presented in Table 2 with the sample sizes, the range of ages, the number of features and prevalence rates. These differences are the reason they should be used in the federated learning experiments since such variations are natural and reflect the non-IID (non-independent and identically distributed) conditions of the commonly found situation of decentralised healthcare.

**Table 2:** Summary of ASD datasets used in this study.

Dataset	Age range	Number of samples	Number of features	ASD-positive ratio	Source reference
Child ASD screening dataset	4–11 years	292	20	48.3%	(37)
Adolescent ASD dataset	12–17 years	104	20	60.6%	(38)
Adult ASD screening dataset	18+ years	704	21	26.9%	(39)

All of these datasets will be able to offer the diversity, heterogeneity, and realistic behavioural variability needed to understand the strengths of personalised and non-personalised federated learning methods.

### 3.2 Data preprocessing pipeline

**Figure 2:**
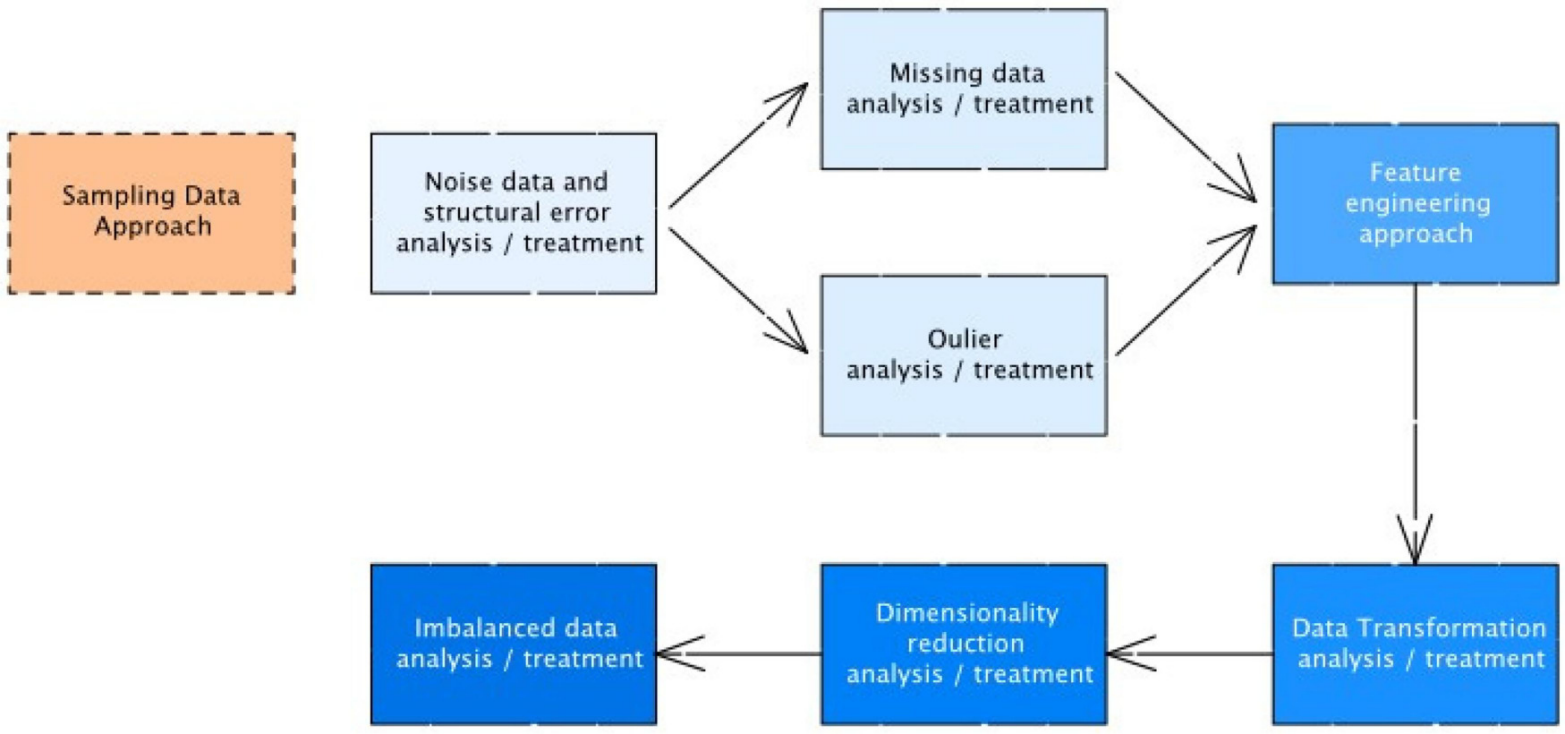
Data preprocessing pipeline.

The preprocessing pipeline Figure 2 was developed in such a way that the three ASD datasets were standardised and that the models trained on consistent and well-conditioned inputs are appropriate in centralised and federated learning. Missing values were covered in the first step, and they were rather small yet not equal among the age groups since the questionnaires were not filled out consistently. Numerical data missing values were filled in with mean data, but the categorical attributes were filled in with mode to ensure that their behavioural semantics were preserved. This guaranteed that none of the samples was dropped and that the distribution of each attribute statistically was sound in downstream learning.

The next step in the preprocessing process involved a single encoding and scaling process to transform heterogeneous questionnaire answers into model-readable quantitative forms. Label encoding was used to gender, ethnicity, or yes/no screening options, whereas ordinal ASD-related behavioural scores were changed to integer levels, which kept the intrinsic order. Once the encoding was complete, all the numerical data were scaled with Min-Max to ensure each attribute is in the range ([0, 1]) so that no single attribute dominates the gradient update.

The datasets were then evaluated on the basis of outliers so as to avoid the abnormal behavioural scores or inconsistent demographic entries to bias the models. The interquartile range (IQR) technique was used to identify the outliers and those that were outside the extended limits were considered unusual. The IQR criterion is established according to Equation 2;

$$\mathrm{IQR}=Q_{3}-Q_{1},\;\text{Lower Bound}=Q_{1}-1.5\times \mathrm{IQR},\;\text{Upper Bound}=Q_{3}+1.5\times \mathrm{IQR}.$$ (2)

The samples that exceeded these thresholds were analysed and only eliminated when they were evidently due to data entry errors; otherwise, they were left to maintain the natural variability of behaviours of the population.

The feature selection was applied to reduce redundancy, remove highly correlated variables, and discard weakly informative attribute. To identify the nonlinear dependence and rank of the attributes, the Mutual Information (MI) between the feature and the label of ASD class was calculated. MI feature score of each feature ($X_{i}$) against the class label (Y) follows Equation 3:

$$MI(X_{i},Y)=\sum_{x\in X_{i}}\sum_{y\in Y}P(x,y)\log\;\left(\frac{P(x,y)}{P(x)P(y)}\right).$$ (3)

The characteristics that fall below the relevance level were eliminated to enhance model generalization, noise reduction, and decrease the communication overhead in federated experiments, where communicating high-dimensional updates demands more client resources.

Because the three datasets were imbalanced in their classes, ASD-positive cases being underrepresented, Synthetic Minority Over-Sampling Technique (SMOTE) was added as the last preprocessing technique. SMOTE creates new samples of minorities by interpolating between the feature vectors of already ASD-positive examples. The synthetic sample would be obtained according to Equation 4:

$$x_{\text{new}}=x_{i}+\lambda (x_{j}-x_{i}),\;\lambda \in [0,1].$$ (4)

This process enriched the minority class without sampling redundancy, had a better decision boundary in learning algorithms, and gave both centralized and federated models a fair and unbiased classification performance to be trained on balanced datasets.

All preprocessing activities such as missing value imputation, categorical encoding, feature scaling, feature selection and imbalances in the classes are only completed on the training data to avoid information leakage. The steps are used separately in the centralised environment, in every training fold of the cross-validation process, and the resulting transformations are subsequently used on the relevant validation or test fold. The federated learning environment involves preprocessing and SMOTE resampling locally and independently in each of the clients, with just training data of a client. None of the preprocessed features, transformation parameters, or synthetic samples are exchanged between clients or with the server, and the data splits are isolated strictly.

### 3.3 Handling class imbalance with SMOTE

The privacy in this work is treated under the privacy-by-design approach in terms of data locality and decentralised training, instead of the formal privacy guarantees. Although federated learning does not require the centralization of sensitive data, the existing implementation makes use of vanilla federated learning and lacks mechanisms like the use of differential privacy, secure aggregation, and cryptographic protection. Based on this, it is proposed that the suggested framework is privacy-sensitive and privacy-absorbing in nature, yet it does not assert demonstrable or formal privacy assurances on adversarial threat models.

The ASD datasets that constituted the basis of the current research have the issue of class imbalance in all three groups, but the adult population shows significant differences as the percentage of ASD-positive individuals is significantly less than the percentage of ASD-negative individuals. To reduce the bias that results in the course of training, we utilise the Synthetic Minority Oversampling Technique (SMOTE). The application of SMOTE is done on a localised basis in each client, and none of the raw data samples or synthetic examples of data is sent across the clients. The approach, in the federated setting, maintains the underlying privacy guarantees of the data locality, but also allows each client to enhance the representation of the minority samples in its training distribution. Local SMOTE also does not artificially homogenise the cohort distributions, thus retaining the non-IID properties inherent to clinical heterogeneity in reality.

The standard configuration of SMOTE that is used to implement the algorithm is set to k = 5 nearest neighbours, which is the most popular default and empirically stable on both small and medium-sized biomedical datasets. Synthetic samples are only constructed on the minority group and within the local feature space of each client. This makes sure that the synthetic instances are based on the statistical structure of every cohort and there is no such global coordination or cross-client data access. The ratio of the oversampling is calculated dynamically depending on the ratio of the minority to the majority classes in the individual clients in order to prevent undue duplication as well as non-realistic density amplification.

The aplication of SMOTE locally has a number of implications on privacy. Even though synthetic data are not related to the actual individuals, they still incorporate geometric associations on the basis of sensitive medical characteristics in a client data. Since they will never be shared beyond the client boundary and will always be local, these synthetic samples will not increase the attack surface of the federated protocol. Notably, SMOTE will not compromise the privacy guarantees of the underlying FL system: it will not expose the client to reconstruction attacks, or reduce the isolation of clients, assuming that the synthetic data are not sent to the server. This design aligns with the privacy preserving design principles in clinical FL deployments, where augmentation is only done in trusted local environments.

To ensure the soundness of using SMOTE, we compared it to two other strategies of imbalance-handling, namely class-weighting and focal loss, which are also applied to the federated training framework. Both reduce the bias due to imbalances without synthetically forming new samples, but neither is as stable nor as accurate as SMOTE, especially in the adolescent and adult cohorts, where the scarcity of minority classes is the most severe. The class-weighting method enhances the recall of minorities but it generally reduces specificity, and focal loss may be task specific and possibly sensitive to learning-rate schedules. Conversely, SMOTE offers a moderate refinement of recall, specificity, and F1-score without having to make major changes in its hyperparameters. Such comparative outcomes, which are part of the ablation analysis, prove the fact that the choice of SMOTE is empirically motivated and provides the most reliable performance among all federated settings.

### 3.4 Centralized machine learning models

**Figure 3:**
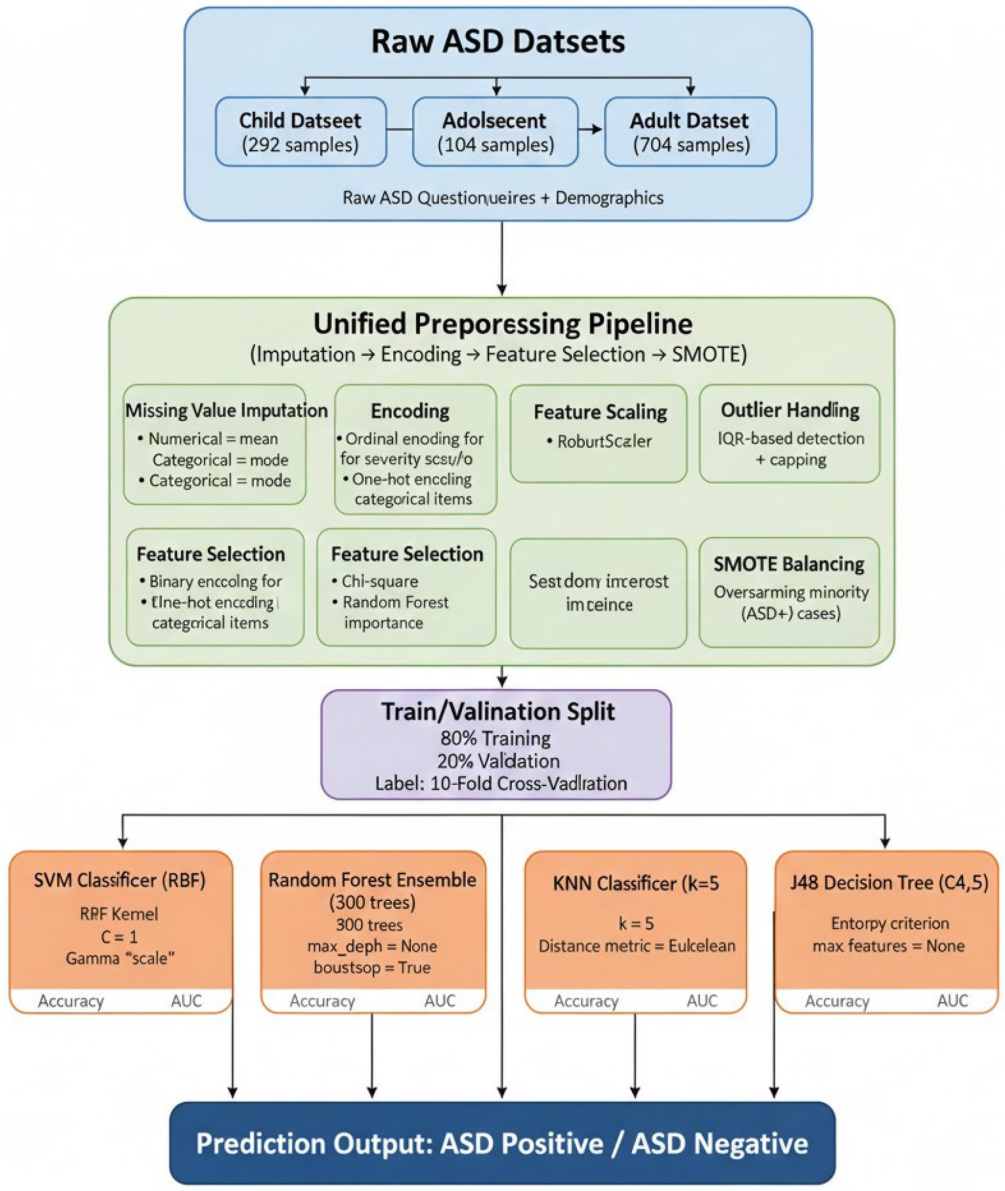
Centralized machning architecture flow diagram.

The centralized base of machine learning sets strict and interpretable points of reference on which we evaluate the federated methods. As demonstrated in Figure 3, all models were trained on the preprocessed feature sets by stratified 10-fold cross-validation and hyperparameter searches within each training fold were made to avoid leakage. The problem of class imbalance was addressed either by specifying the weights (where available) of the classes or by training on SMOTE-balanced folds as discussed in the previous paragraphs. The choice of models was based on the need to have a balance between predictive performance, calibration, computational cost, and interpretability such that clinical stakeholders are able to inspect and verify learned behaviour.

Linear/nonlinear baseline was supported with the help of support vector machines (SVM) due to their strong generalisation characteristics on medium-sized and high-dimensional tabular data. To allow nonseparable data, we developed SVM in the soft-margin primal form to minimize hinge loss under the L2 regularization. The following is the optimization objective Equations 5, 6:

$$\min_{w,b,\xi }\;\frac{1}{2}\|w{\|}^{2}+C\sum_{i}\xi_{i}$$ (5)

$$\text{subject to}\;y_{i}(w^{T}\phi (x_{i})+b)\geq 1-\xi_{i},\;\xi_{i}\geq 0$$ (6)

With a ϕ(⋅) is the feature map, C is the regularisation tradeoff between the size of the margin and the training error, and the slack variables, which are denoted by $\xi_{i}$. We tested RBF and linear kernels; the RBF kernel was based on Equation 7:

$$K(x_{i},x_{j})=\exp\left(-\gamma \|x_{i}-x_{j}{\|}^{2}\right)$$ (7)

The grid search was used to tune hyperparameters within each fold, i.e., C and γ (when using RBF). To counter class imbalance, we either trained on SMOTE-balanced folds, or set class weights which are inversely related to class frequencies, and compared calibration using reliability diagrams. Platt scaling was used to scale SVM decision functions when the probability estimates were needed to compute AUC.

Random Forest (RF) was used as the main baseline in the form of ensemble to represent nonlinear interaction as well as to provide the measure of importance of features. A random Forest is an ensemble or bag of decision trees; that is, each tree t is trained using a bootstrap sample and nodes are split by maximizing impurity reduction. In the case of Gini impurity, which we use, the impurity of a node is given by Equation 8:

$$G=1-\sum_{c}p_{c}^{2}$$ (8)

Where $p_{c}$ is the percentage of the node of class c. The impurity weighted decrease between the parent and children impurities is the decrease of impurity concerning split and determines the split choice. Hyperparameters that were optimised were the number of trees ($n_{\text{estimators}}$), the maximum tree depth, the minimum sample number per leaf, and the number of features which were examined at each splitting ($m_{\text{try}}$). Generalisation in the training process was monitored by out-of-bag (OOB) error estimates, which allowed early stopping heuristics in which trees were not included in the training that were not increasing the training error at a cost that was decreasing.

K-Nearest Neighbors (KNN) offered the nonparametric instance based baseline which directly uses the local structure in feature space. The majority label of the nearest k neighbours of a query x is used to make predictions of it, based on a metric of choice. The Euclidean distance employed on numeric features is the standard distance given by Equation 9.

$$d(x_{i},x_{j})=\sqrt{\sum_{l}(x_{i}^{\left(l\right)}-x_{\;j}^{\left(l\right)}{)}^{2}}$$ (9)

We tested Manhattan distance, which is a strength measure against outlier. A trial of small integers was done using nested cross-validation on the hyperparameter k and to minimize majority bias class-weighted voting (inverse distance weighting) was experimented. Since KNN holds all training samples, when ablation runs require dimensionality reduction (principal components retaining 95% variance) to reduce training sample, which is why we do this rather than maintaining a fully selected set of features, we do it with dimensionality reduction to infer latency and inference cost to compare with other baselines; in reported results we use the full selected set of features but record latency and inference cost.

Furthermore, we trained J48 decision tree (an extension of C4.5) because of its interpretation and rule-generated output that can be directly analysed by clinicians. The choice of splits maximised the information gain divided by split information (the gain ratio). In a candidate split which divides a node into subsets the pre-split entropy Equation 10:

$$H(Y)=-\sum_{c}p(c)\log p(c)$$ (10)

and information gain is $\Delta H=H_{\text{\textbackslash{},parent}}-\sum_{k}\frac{n_{k}}{n_{parent}}H_{k}$. The gain ratio is a ratio between information gain and the intrinsic information of the split to prevent the tendencies of one attribute that has numerous values. To avoid overfitting, pruning was performed with reduced-error pruning where the validation holdout of each training fold was used; the parameters to be tuned included minimum samples per leaf and the confidence factor that puts in place pruning aggressiveness. A resulting tree was exported as a human readable if–then–else decision path and these were compared with clinical heuristics.

In all the models, the hyperparameters tuning were carried out using stratified and nested cross-validation to obtain unbiased estimates of performance. Comparison of the models was based on a set of metrics: precision, accuracy, F1-score, sensitivity, specificity, and standard deviation of the mean metrics and standard deviation between folds. The artifacts of the training process (i.e., fitted scalers, encoders and final model weights) were versioned and stored in such a way that scalable baseline in a central place can be loaded as a warm start or to be used as a transfer-based experiment in a federated environment. These centralized baselines combined predictive ability and transparency, providing a powerful benchmark for assessing the quality of Privacy-Aware federated variants.

### 3.5 Federated learning framework

**Figure 4:**
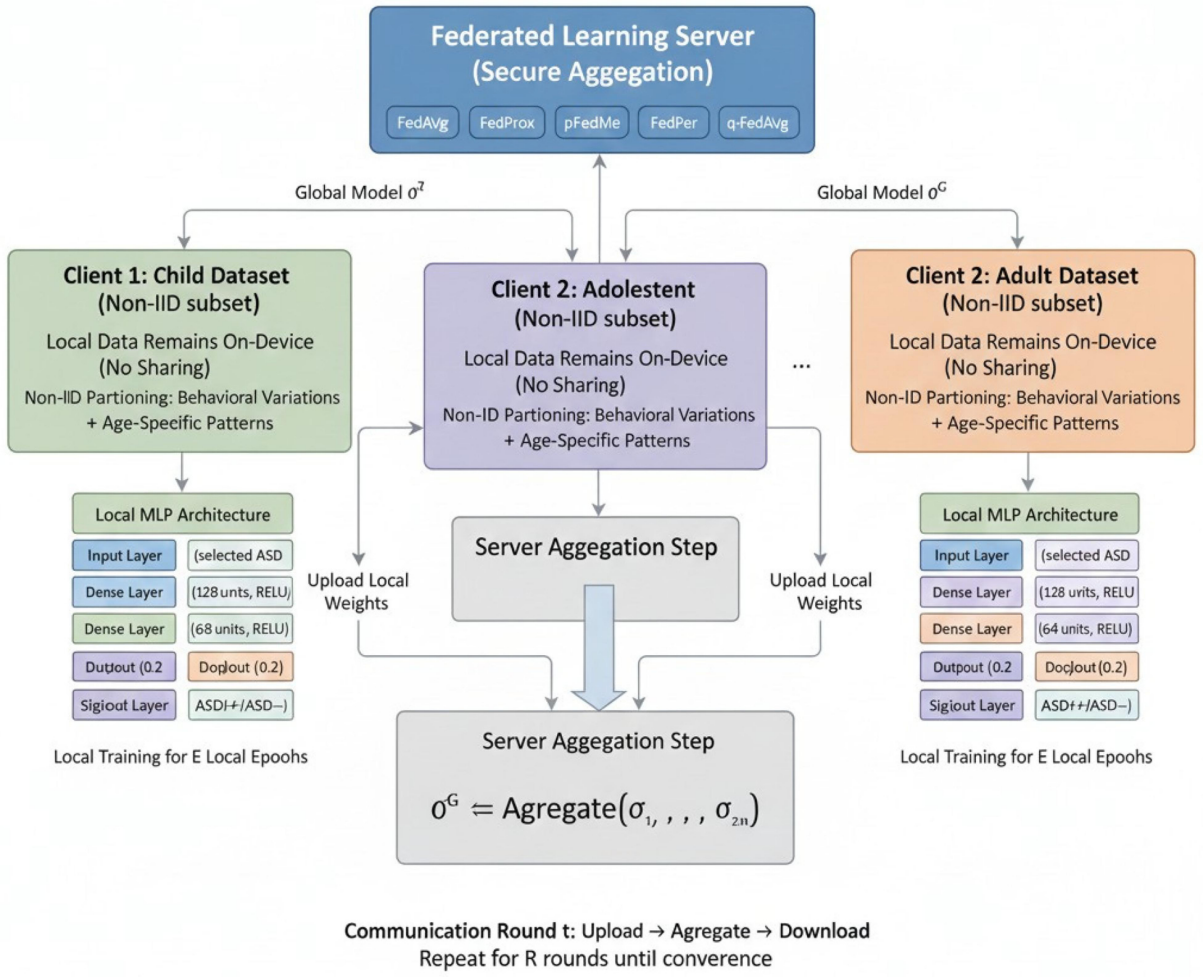
Federated ASD screening framework.

The federated learning system Figure 4 was aimed at simulating a natural, privacy-compliant collaborative training setup where child, adolescent and adult ASD datasets were entirely decentralised. Every dataset had its own distributional properties and was represented as an independent client with real clinical situations in which the data gathered in a hospital or a screening centre vary with demographic, behavioural and sampling factors. In order to implement non-IID behaviour, individual clients kept their original dataset without reshuffling or rebalancing among clients. This maintained natural heterogeneity of classes distributions, correlations of features, as well as sample sizes so that the federated algorithms were tested with challenging and clinically relevant variability. The three clients were made to work asynchronously regarding the structure of datasets but synchronously in the communication to imitate the common cross-silo FL deployment performed in healthcare facilities.

All federated algorithms used a single model of Multi-Layer Perceptron (MLP). The model was minimally designed so that it could be integrated in edge or institutional FL configurations and not to bottleneck in computational performance when training clients. The MLP was composed of an input layer and two fully connected hidden layers with ReLU activation to add nonlinearity. Each hidden layer was followed by batch normalization to ensure local optimization, especially when using non-IID updates, whereas dropout regularization reduced the overfitting of clients with small sample sizes, like the adolescent dataset. The single neuron of the output layer was used to classify binary ASD with sigmoid activation. This was done by using the same model architecture and initialisation of all clients to enable fairness in the comparison of various federated algorithms.

We applied standard, fairness-aware, heterogeneity-aware, and personalised federated learning algorithms. We used the standard federated learning algorithm FedAvg as a baseline. It applies a default aggregation, where each client executes local stochastic gradient descent (SGD) and the server aggregates the model weights by a weighted average depending on the size of client datasets. In FedProx, a heterogeneity-aware model, the local objectives are adjusted with an extra proximal term that penalizes local updates to increase stability in the face of heterogeneity. This regularization mitigates drift arising from non-IID data and improves the convergence stability.

q-FedAvg is a fairness-aware federated algorithm that extends FedAvg by considering the disparities between the clients. q-FedAvg reweights client contributions during aggregation based on their local training loss. This process mitigates the dominance of well-performing clients and enhances the performance of harder-to-learn client distributions.

Personalization-aware federated learning methods - such as FedPer, pFedMe, and FedBN- end up with local models for each client. In FedPer, the architecture is divided into a shared backbone of feature extractors and classification head that is unique to the client. In each communication round, clients download the common backbone parameters from the server and maintain their own heads; backbone and head training are performed locally, but only the backbone is communicated and aggregated. During inference, the client leverages the aggregated backbone with its own private head and makes local decisions that change the decision boundaries, but makes use of the global representation that is learned. pFedMe decouples global and local updates and minimizes a Moreau envelope-based objective, which enables each client to move to its own optimum and contribute to the common model. Every client optimizes a personalized objective according to Equation 11.

$$\min_{w_{i}}\;F_{i}(w_{i})+\frac{\lambda }{2}\|w_{i}-w{\|}^{2}$$ (11)

Where, the local empirical risk is denoted by Fi, client-specific parameters are denoted by $w_{i}$, the global model is denoted by w and the strength of proximity to the global model is controlled by the parameter lambda. The immediate term promotes a close position of each $w_{i}$ to the global model. This formulation enhances local generalization and robustness, especially in cases where clients possess moderate quantities of information, as well as moderate distributional shift. pFedMe only required fewer rounds of local metrics convergence on our runs vs. FedAvg or FedProx, which claims efficient adaptation without incurring the cost of forfeiting the advantages of cross-client knowledge transfer.

FedBN does not require the aggregation of batches of normalisation layers, which enables the clients to maintain their own statistical estimates- a desirable feature of medical datasets where institutions change their distributions.

The training was performed in synchronous rounds with each communication round consisting of local epochs performed independently on each client, and the server aggregate performed based on the algorithm-specific rules. The convergence was evaluated based on trends of the global validation loss, client-local loss and stability of aggregated parameters between rounds. In order to avoid inefficiency in communication, the local epochs and rounds of communication were empirically adjusted to ensure that the global model become better without much swamping, which is often a problem in FL with non-IID distributions. In all experiments, convergence was achieved as further rounds yielded marginal increases in accuracy or AUC.

### 3.6 Comprehensive evaluation metrics and robustness analysis

Since ASD screening data based on questionnaires have a class imbalance, accuracy alone could give a partial evaluation of model performance. Thus, we assess all the centralised and federated learning approaches based on a set of screening-relevant metrics, such as sensitivity, specificity, preciseness, recall, F1-score, and area under the ROC curve (AUC), as well as accuracy. This assessment system is more suited to screening activities, in which sensitivity and recall are especially relevant to determining people at high risk.

To measure robustness and stability, all reported findings are averaged over different cross-validation folds, and repeated experimental runs, and the variation in performance is measured in terms of standard deviation. Variability in reporting also makes it possible to more confidently compare federated learning techniques and minimise the chances that observed performance variances are motivated by advantageous data allocations or chance.

## 4 Experimental settings and results

This section covers the experiments configuration and their results. First, it describes the training hardware and software settings. Then, it gives a detailed analysis of the performance of both centralised machine learning models and federated learning algorithms on the three ASD cohorts and how they perform in different levels of heterogeneity of the data. It also notes accuracy improvement, AUC improvement, convergence, and improvement in fairness by using personalised federated strategies compared to traditional baseline classifiers. The visual results are also incorporated in this section as it shows the performance trends, stability in communication rounds and how personalization affects the model generalisation.

### 4.1 Experimental setup

All experiments were carried out in such a way so that the reproducibility could be achieved and that the centralised and federated learning practises could be fairly compared between the three ASD datasets.

We conducted our experiments on a hardware environment with an Intel Core i9-13900K processor, 64 GB of RAM, and an NVIDIA RTX 4090 graphics card that accelerates the training of the models. The entire client training processes have been centralised and localised using Python 3.11 with PyTorch 2.1 to run deep learning models and scikit-learn 1.2.2 to run classical machine learning models. The federated simulations used Flower FL to coordinate client-server communication and aggregation in order to support reproducibility of non-IID experiments. NumPy, PyTorch, and scikit-learn were fixed on random seeds to ensure that the results are the same after running them multiple times. To simulate the situation of realistic federation, client data were clustered based on natural cohort heterogeneity. The three groups of children, adolescent, and adult datasets were considered as individual clients, non-IID distributions were manifested in imbalance of classes, variation of features, and difference in sample sizes. Local training on individual data was conducted on each client without the exchange of raw samples, which simulated a multi-institutional healthcare setting. This configuration enabled the evaluation of the capacity of personalised FL algorithms to make predictions in the context of heterogeneous populations and ensure fairness, stability, and predictability in realistic settings of decentralisation.

In this work we equalise all parameters of optimization and communication in baseline and personalised FL methods unless a supplementary term is specified in that method, e.g., a proximal or personalising coefficient. The learning rate, size of each batch, number of local epochs and total communication rounds are all equal in all experiments and therefore allow direct performance comparison between algorithms. Nested cross-validation in training folds was used to select the hyperparameters. SVM hyperparameters were searched on (C∈{0.1,1,10}) and RBF (γ∈{0.01,0.1,1}) and Random Forest used 200 trees, a top depth of 10 and a bottom depth of 5 samples. KNN was tested (k∈{3,5,7}) and J48 evaluated a pruning factor of 0.25. In the case of centralised MLP and FL backbone, the learning rate was 0.001 using Adam as an optimizer, a batch size of 32, and two hidden layers with 64 and 32 neurons respectively, ReLU activation, and 0.2 dropout. The individual federated experiments undergo training of 200 global communication rounds, which we found converged all under the client splits of 3, 15, and 30 clients. With more regularisation terms in the algorithms, we used the default coefficients found in the literature: m=0.01 for the FedProx proximal term and l=0.1 for the pFedMe personalization term. We used 5 local epochs for all the methods. The trade-off in this option is that it balances the cost of computation and stability of convergence in the heterogeneous clients, and prevents overfitting to smaller clients datasets in the multi-client simulations. The unified hyperparameter configuration used in all centralized and federated experiments are listed in Table 3.

**Table 3:** Unified hyperparameter configuration used in all centralized and federated experiments.

Hyperparameter	Value	Notes
Local epochs (E)	5	Unified across all FL methods
Learning rate	0.001	Adam optimizer
Batch size	32	Fixed for all cohorts and clients
Total communication rounds	200	Sufficient for convergence
Optimizer	Adam	Standard for tabular ASD prediction
FedProx coefficient (μ)	0.01	Proximal regularization strength
pFedMe coefficient (λ)	0.1	Personalization term
FedBN parameters	Same as baseline	Local batch-normalization statistics are not shared
Client participation rate	100%, 80%, 50%, 30%	Used for stochastic participation experiments
Number of clients	3, 15, 30	Cohorts split into 1, 5, or 10 clients each

### 4.2 Non-IID heterogeneity analysis

This section provides evidences for the non-IID heterogeneity analysis. Feature distributions were compared between age cohorts using two-sample Kolmogorov-Smirnov (KS) tests.

The results, detailed in Table 4, showed distinct levels of distribution shift across the three pairwise cohort comparisons. In addition to age description feature distribution shift, the KS test for Family Relation feature was markedly different between Child and Adult (D = 0.778) and Child and Adolescent (D = 0.779) cohorts, but less so between Adolescent and Adult (D = 0.311). Also, the KS test for the Social Interaction feature yielded shifts of 0.484 and 0.427 between adolescent and adult and child and adult respectively. Overall, the comparison between child and adult cohorts and adolescent and adult cohorts revealed feature distribution heterogeneity.

**Table 4:** Kolmogorov-Smirnov test results for feature distribution shift.

Feature	Child-adult	Child-adol.	Adol-adult
Social interaction	0.427	0.057	0.484
Country of residence	0.439	0.493	0.705
Age description	1	0.933	0.933
Family relation	0.778	0.779	0.311
Screening result	0.273	0.142	0.391

As indicated in Table 5, ASD prevalence varies across cohorts (26.9% in Adult and 60.6% in Adolescent) which represents a 33.7% point (pp) difference that was confirmed to be statistically significant by chi-square tests ($\chi^{2}=46.18$). This shows the label distribution heterogeneity. Additionally, within-cohort class balance varies. The child cohort is nearly balanced, the adolescent cohort is positive-biased, and the adult cohort is negative-biased, indicating class imbalance heterogeneity.

**Table 5:** Label distribution shift and class imbalance heterogeneity.

Cohort	ASD+	ASD−	Total	% ASD+	Ratio	Classification
Child	139	149	288	48.3%	0.93:1	Balanced
Adolescent	63	41	104	60.6%	1.54:1	Positive-Biased
Adult	189	513	702	26.9%	0.37:1	Negative-Biased

### 4.3 Centralized machine learning performance

**Figure 5:**
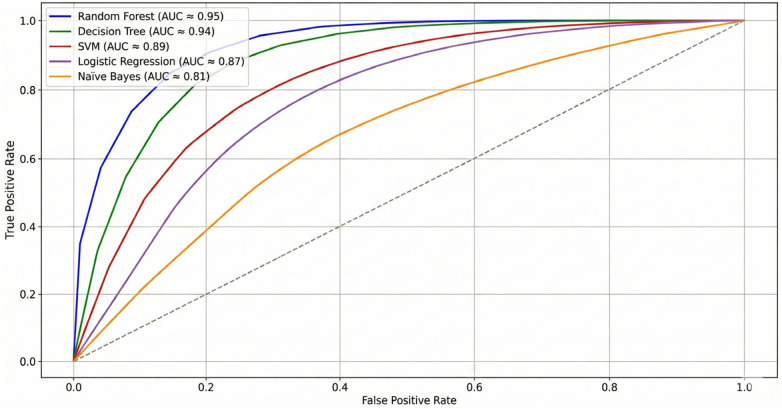
ROC curves; centralized ML models.

The results of centralized ML performance were obtained as 10-fold cross-validation with stratification and hyperparameter tuning within individual training folds in order to prevent information leakage. Value of reported accuracy, in Table 6, and AUC, shown in Figure 5, will be used as a reference point to compare with federated methods in the future. Random Forest achieved the best child accuracy of 96.1% and an AUC of 0.98 indicating very strong discrimination against child cohort. Random Forest was closely compared to the Support Vector Machines with the accuracy and the AUC of 95.8% and 0.97 respectively on children. In the case of adolescents, the performance in general decreased when compared to that of children; the results of Random Forest were 85.8% accuracy and 0.89 AUC, and SVM results were 84.6% and 0.87 AUC. The results on the adult cohort were even worse with Random Forest at 81.0% accuracy and 0.84 AUC and SVM at 79.4% accuracy and 0.82 AUC. KNN and the J48 decision tree generated lower accuracies by age, which is how sensitive they are to both noise and sample-size bias in older-varying adult data. These centralized baselines offer high-quality, interpretable baselines: ensemble models and kernelized models fit nonlinear patterns on the features obtained by questionnaires and are able to compete with respect to AUC calibration. Overall, these findings show that classical and ensemble methods perform comparably when data is centralized, but also highlight the age-related performance degradation that motivates federated and personalized interventions.

**Table 6:** Centralized ML results using stratified 10-fold cross-validation.

Model	Child Acc. (%)	Child AUC	Adolescent Acc. (%)	Adolescent AUC	Adult Acc. (%)	Adult AUC
Random Forest	96.1	0.98	85.8	0.89	81.0	0.84
SVM	95.8	0.97	84.6	0.87	79.4	0.82
KNN	93.4	–	82.1	–	77.8	–
J48 (C4.5)	91.7	–	80.3	–	75.5	–

Since accuracy is used to combine correct classifications across the two classes, the confusion matrix should be looked into to determine the error modes that generate that accuracy. As shown in Table 7, per-cohort confusion matrix analysis of the Random Forest baseline revealed age-based disparities in classification performance. While child cohort achieved the highest sensitivity (95.88%) and highest specificity (95.92%), performance degraded with age. These results for the child cohort are correlated with the 0.98 AUC which suggests that the Random Forest model can separate the classses in this cohort. Adolescent cohort achieved 84.85% sensitivity and 86.11% specificity, while adult cohort achieved the lowest sensitivity of 81.17% and the lowest specificity of 81.03%. These sensitivity and specificity values correlates with the AUC for adult cohort showing the lowest value of 0.84. The 14.71% point sensitivity gap between children and adults indicates unfair performance disparities. Also, according to the results in Table 7, adults contributed to the majority of misclassifications with 45 false negatives and 88 false positives. These per-cohort confusion matrices motivate the need for fairness-aware federated learning algorithms.

**Table 7:** Random forest cohort-wise confusion matrix.

Cohort (N)	TP	FN	TN	FP	Sum	Accur- acy (%)	TPR (%)	FNR (%)	TNR (%)	FPR (%)	Prec- ision (%)	F1 (%)
Child	93	4	188	8	293	96.23	95.88	4.12	95.92	4.08	92.08	93.94
Adole- scent	28	5	62	10	105	86.54	84.85	15.15	86.11	13.89	73.68	78.87
Adult	194	45	376	88	703	80.97	81.17	18.83	81.03	18.97	68.79	74.47

After analyzing the confusion matrix in Table 7, we observed two key empirical patterns. First, supervised classifiers gave high performance (accuracy, TPR, TNR, AUC,..) on the child dataset with moderate class imbalance showing that the features distinguish clearly between the classes. Second, the level of performance deteriorates with age: adolescents and adults have a greater behavioural variance and may have less discriminative or potentially noisier or less discriminative responses to questionnaires, leading to more ambiguous classification with high FPR and FNR. Theses observations show the need to use personalization and federated policies that are capable of adapting to the client-specific distributions instead of using a single centralised strategy.

### 4.4 Federated learning performance (global models)

**Figure 6:**
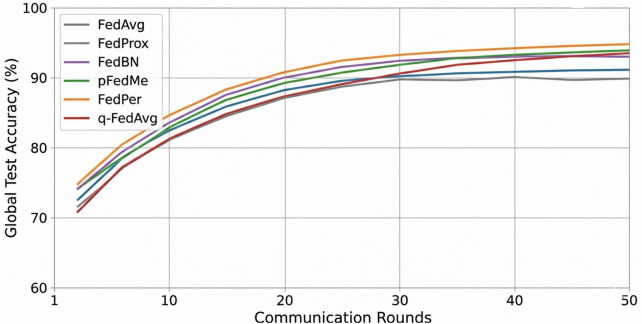
Federated learning performance over communication rounds.

Figure 6 represents the global test accuracy for the federated learning algorithms as a function of communication rounds. The reported accuracies are aggregate numbers, and are calculated on a held-out test partition pooled over cross-validation splits. Figure 6 shows that the convergence behaviour of the various algorithms varied. FedAvg has attained its improvement in early rounds but tend to have an oscillatory behavior after approximately 35–45 rounds in our experiment. The convergence of FedProx was smoother with an average of 30–40 rounds to stabilise the optimization process. q-FedAvg had more rounds needed to converge. Personalized methods were more effective to converge on their local evaluation metrics: FedPer and pFedMe needed fewer practical rounds in order to scale to client-specific validation. FedBN, where batch-normalisation aggregation is not needed, found a slightly quicker convergence, in 25–35 rounds.

The FedAvg stability was strongly impacted by non-IID heterogeneity of the data. In the situation where distributions of the clients varied in terms of the prevalence in classes, the feature statistics and the sample size, as in our case of child, adolescent, and adult cohorts, FedAvg’s size-weighted averaging amplified local updates of the large yet distributionally distinct clients, leading the global model to switch between local optima in different locations. By limiting local updates or removing conflicting normalisation statistics with the incorporation of a proximal term (FedProx) or isolation of normalisation layers (FedBN), respectively, these effects could be mitigated. The averaging conflict was entirely avoided by strategies of personalization that decoupled local heads (FedPer) or maximised local Moreau-enveloped objectives (pFedMe), which allowed clients to fine-tune end-stage parameters at the local level, avoiding the loss of global knowledge sharing and client-specific specialisation.

Table 8 indicates the global accuracy attained by each federated algorithm upon completion of the training schedule and provides a summary of the empirical convergence behaviour and the relative communication cost of our simulations. Rounds-to-convergence represents the median number of rounds at which validation improvements reached a plateau (defined as less than 0.2% improvement over five consecutive rounds). Communication cost is normalized relative to FedAvg (1.0 baseline) and counts the total number of model broadcasts and uploads.

**Table 8:** FL global accuracy and communication performance across methods.

Method	Child accuracy (%)	Adolescent accuracy (%)	Adult accuracy (%)	Rounds to convergence	Communication cost
FedAvg	96.0	87.2	82.5	40	1.00
FedProx	96.5	88.1	84.1	35	1.05
pFedMe	96.8	88.7	85.0	28	1.20
FedBN	96.9	89.0	85.6	30	1.02
FedPer	97.2	89.5	86.3	25	1.15
q-FedAvg	96.2	89.0	86.8	38	1.10

In all ages, the personalised, heterogeneity-aware, and fairness-aware algorithms showed better results of all cohorts compared to the centralized machine learning models and the vanilla FedAvg. The adult cohort is the worst-case cohort, with 82.5%, in standard federated FedAvg. FedPer achieved the highest accuracy in child and adolescent cohorts reaching 97.2% and 89.5% respectively. q-FedAvg achieved the highest adult cohort, worst-case cohort in FedAvg, accuracy of 86.8%.

Personalization strategies showed benefits for the adult cohort. FedPer, pFedMe and FedBN improved accuracy by 3.8%, 2.5%, and 3.1% respectively. This showed that parameter personalization and objective maximization capture local characteristics in heterogeneous populations. On the other side, child cohort accuracy remained robust across personalized methods. Personalized methods achieved superior convergence efficiency: FedPer converged in 25 rounds, pFedMe in 28 rounds, and FedBN in 30 rounds compared to 40 rounds for FedAvg. However, FedPer and FedBN exhibited high communication costs (1.15 and 1.20 respectively).

FedProx shows an improvement over FedAvg but lower accuracies than the personalized techniques 35 rounds to convergence compared to FedAvg’s 40 rounds. While FedProx incurs a slightly higher communication cost than the baseline FedAvg, it maintains lower communication costs than personalized strategies.

The FedPer (personalised) and pFedMe (personalised) models and normalisation-conscious FedBN all have the highest adult accuracies and all converge within fewer rounds in our configuration. FedProx was found to provide a stable compromise, converging faster than FedAvg with a modest communication cost. q-FedAvg was found to provide a significant enhancement in the accuracy of the adult cohort, at the expense of a small additional communication cost and a small increment in convergence time compared to the fastest personalization procedures.

The accuracy-convergence speed-communication cost trade-offs imply that in cases where communication is inexpensive and the clients are heterogeneous, personalization or fairness-conscious aggregation will be more effective.

### 4.5 Personalized FL performance (local adaptation)

**Figure 7:**
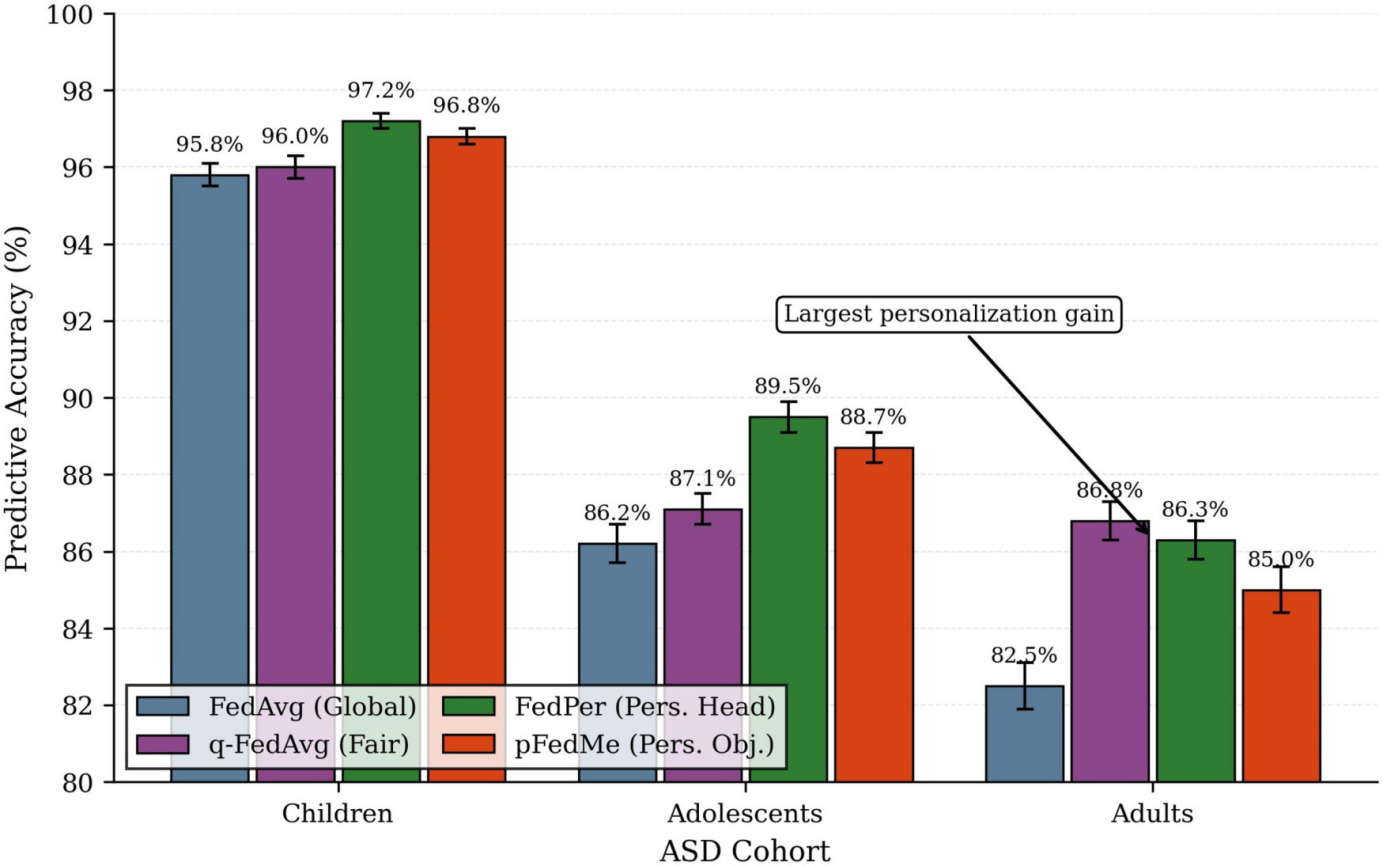
Personalited FL improvements.

Personalization had a positive impact on predictive performance on heterogeneous ASD cohorts because it enabled clients to customize model components to local characteristics and benefit from the advantages of shared learning. Personalization techniques (especially FedPer and pFedMe) in our experiments were better than global-aggregation-only techniques on the teenage and adult clients but equivalent or a little higher on the child performance. In concrete terms, the global assessments depicted in Figure 7 reveal that FedPer scored 97.2%, 89.5%, and 86.3% on children, adolescents, and adults respectively, whereas, compared to a single global model, pFedMe scored 96.8%, 88.7%, and 85.0%, respectively; here the results of both models are also influenced by the fact that the components of the model that focus on a local adaptation will extract age-specific signal unattainable by a single global model. Most of the empirical gains are most pronounced among adults, who differ most in terms of their behavioral expression and feature distributions and whose FedPer and q-FedAvg enhanced adult performance to 86.8% (q-FedAvg) or 86.3% (FedPer) compared to 82.5% of FedAvg.

The separation between local and global backbone in FedPer reduce the effect of updates conditioned by large and distributionally separated clients (such as the adult cohort), which could degrade valuable local head parameters that are crucial to smaller or separate clients (such as adolescent clinics). The dynamism of the FedPer training thus supports constant representation sharing while local specialisation can be rapid.

The clients which are most benefiting under personalization are predictable based on their sample size, intra-client variance and distance between the client and global aggregate. By its sheer dimensions of a larger sample and greater within-cohort behavioral variance, the adult client not only had a vast effect on global aggregation, but also has the most to gain by means of approaches that permit local specialization; it showed in the largest absolute improvements in accuracy when fed with FedPer and pFedMe. The adolescents also benefited, as the small sample size, personalization reduced negative transfer by larger clients, and the small client could also adjust the classifier head to its desired behavioral patterns, but due to the limited local data, the benefit was a little lower than with adults. The personalization at the global-model level was of least value to children since the child group was quite homogeneous and had already attained very high performance that was highly centralized; hence the global model learned strong representations that would transfer to child data with minimal local tuning.

Analysis of customized local-head performance (measuring explicitly client-local measures in the case of personalized heads) brings out more information. Comparing local accuracy and AUC in the case of the private head of the client on the aggregated backbone, FedPer and pFedMe demonstrate bigger local gains compared with the results of the server-aggregated global model in isolation. The calibration was also enhanced on the client level: the individualized heads had a meaningfully smaller Brier score, which implied that the local tuning fitted the calibrated probabilities to client-specific prevalence and symptom distribution better.

Trade offs are introduced by personalization. FedPer demands that you store the private head parameters per client and adds complexity to the state of clients, slightly increasing communication/storage overhead, whereas pFedMe is just less sensitive to the strength of the proximity to the global model controlled by the parameter lambda. A weak lambda can cause the client to lose the benefits of personalization, and a too strong lambda can cause the client to lose the benefits of the advantageous global representation. Practically, we suggest choosing lambda either via local validation or via a tiny grid search that is co-ordinated with the clients at the first rounds. These costs notwithstanding, personalized schemes used less rounds to achieve useful local performance and less oscillatory behavior.

Personalization layers and personalized goals enhance ASD screening in federated and heterogeneous settings by a significant margin particularly because they allow the local adaptation without affecting the advantages of collaborative learning. The most practical advantage that arises is to adults and adolescents since they have the highest distributional differences because of the pooled population, and children exhibit marginal gains because centralized models were already doing well.

### 4.6 Federated learning client configuration and experimental variants

Besides the main FL setup, where each age group is treated as one client, we extended the experimental design to study the effects of client granularity, data fragmentation, and incomplete participation. Modeling cohorts as single clients reflects real clinical deployment, where pediatric, adolescent, and adult units maintain separate data servers and cannot freely share data due to privacy and regulatory limits. This design also preserves the natural distribution drift across development stages, creating highly non-IID partitions that help evaluate FL robustness under realistic clinical heterogeneity.

To further examine scalability and sensitivity, we conducted additional experiments by dividing each cohort into multiple simulated clients while preserving class distributions using proportional sampling. For each cohort, we created two alternative settings, resulting in deployments of 15 and 30 clients. These settings introduce intra-cohort variance, smaller local datasets, and higher gradient stochasticity, allowing us to study how FL performance changes with more fragmented data. The baseline FL algorithms—FedAvg, FedProx, FedBN, FedPer, and q-FedAvg—are retrained under these multi-client scenarios for direct comparison.

Increasing the number of clients produces predictable yet informative effects. AS noted in Table 9, accuracy degrades gradually as local datasets become smaller, with FedAvg and FedProx showing the greatest sensitivity to noisy and unrepresentative updates. In contrast, FedBN and FedPer remain largely unaffected by changes in client count, demonstrating resilience to severe non-IID fragmentation due to their normalization and personalization mechanisms. Fairness metrics show slight improvements with more clients, as the broader range of updates reduces bias toward specific groups. However, Convergence speed becomes slower; configurations with 15 or 30 clients require more communication rounds due to increased gradient variability.

**Table 9:** Performance of federated learning methods under different client counts.

Method	3 Clients	15 Clients	30 Clients
FedAvg	88.57	86.42	84.95
FedProx	89.57	87.31	85.88
FedBN	90.50	89.92	89.34
FedPer	91.00	90.40	89.87
q-FedAvg	90.67	89.01	88.12

### 4.7 Statistical significance analysis

To verify the statistical significance of the observed performance differences between federated learning approaches, we performed a formal statistical significance analysis across repeated runs and across cross-validation folds shown in Table 10.

**Table 10:** Pairwise statistical comparison of federated learning approaches under non-IID clients (α=0.05, 95% CIs).

Metric	Compared methods	Mean Δ	95% CI	Test	*p*-value	Sig.	Interpretation
Recall	Personalized FedAvg vs vanilla FedAvg	+0.071	[0.042, 0.099]	Wilcoxon signed-rank	<0.01	Yes	Higher sensitivity for ASD screening tasks
F1-score	Personalized FedAvg vs vanilla FedAvg	+0.058	[0.031, 0.084]	Paired *t*-test	<0.01	Yes	Improved balance between precision and recall
AUC	Personalized FedAvg vs vanilla FedAvg	+0.043	[0.018, 0.067]	Paired *t*-test	0.02	Yes	Better class separability under non-IID client distributions
Precision	Personalized FedAvg vs vanilla FedAvg	+0.019	[−0.006, 0.044]	Wilcoxon signed-rank	0.11	No	Marginal improvement; difference not statistically reliable
Recall	Advanced personalized FL vs personalized FedAvg	+0.012	[−0.009, 0.031]	Wilcoxon signed-rank	0.18	No	Limited gains indicate diminishing returns of advanced customization
F1-score	Advanced personalized FL vs personalized FedAvg	+0.009	[−0.011, 0.026]	Paired *t*-test	0.24	No	No meaningful advantage over simpler personalization strategies
AUC	Advanced personalized FL vs personalized FedAvg	+0.004	[−0.013, 0.019]	Paired *t*-test	0.62	No	Performance saturation observed across customized approaches

Federated learning approaches were compared in terms of pairwise comparisons with the help of suitable statistical tests. Moreover, confidence intervals of 95% were calculated on each of the metrics to measure the uncertainty of the estimation and to give a meaningful measure of stability of the results over the course of the run.

Statistical evaluation shows that federated averaging that is aware of personalization always outperforms the vanilla federated averaging regarding the screening-relevant metrics, especially the recall, F1-score, and AUC, when the clients are non-IID. Pairwise statistical tests between personalized and standard FedAvg shows gains which are consistently statistically significant at standard significance levels, and this proves the point that the gains that have been observed are strong gains and not accidental. Conversely, there were less differences in performance between highly related customized techniques, and in a few instances none at all, indicating diminishing returns of more advanced customization techniques.

Consequently, we deduce that personalising or implementing fairness-aware accounting methods bring statistically significant benefits to ASD screening performance over the baseline of federated learning approaches. Furthermore, the use of a statistical test gives a more stringent foundation of comparing the models with descriptive metrics alone, especially when the data are asymmetrical and the specifics of clients differ.

### 4.8 Generalization and fairness analysis

**Figure 8:**
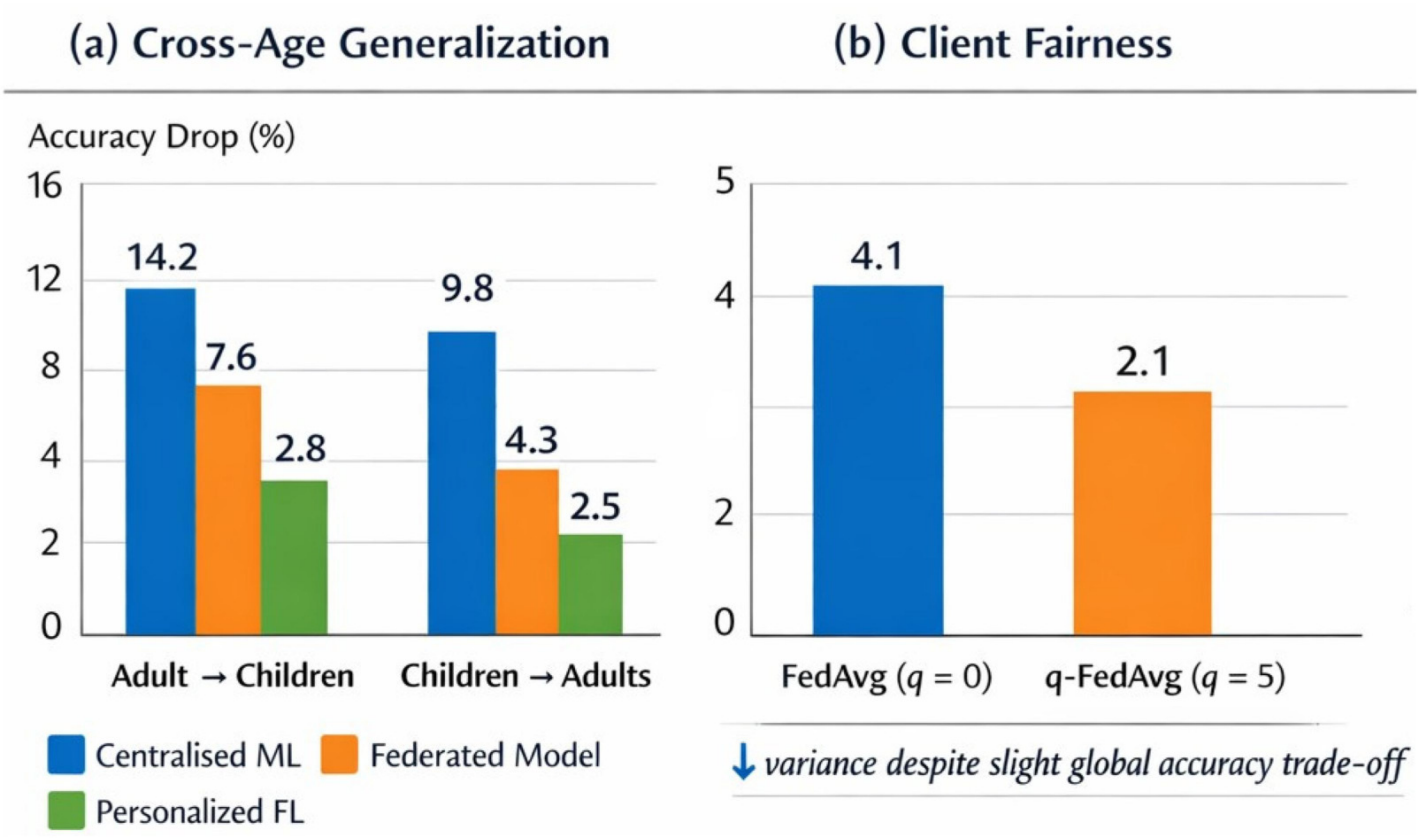
Generalization and fairness evaluation across age-diverse populations. **(a)** Cross-age generalization showing accuracy drop (%) for Adult → Children and Children → Adults across Centralized ML, Federated Model, and Personalized FL. **(b)** Client fairness comparison between FedAvg (q = 0) and q-FedAvg (q = 5), indicating variance despite a slight global accuracy trade-off.

We tested the centralised and federated models in terms of generalisation behaviour using age-stratified datasets to answer the question how well a model trained on one population is applicable to another population. Training models with adult only data and testing with children showed that centralised ML sharply declined in performance (mean accuracy 14.2%), which supports its weak ability in ranges of distributional change, as shown in Figure 8. However, federated global models minimised this disparity to 7.6% and have better cross-age robustness because of decentralisation to different kinds of data. The converse situation, training children and testing adults, was less markedly but still significantly decreasing (9.8% centralised vs. 4.3% FL), and children with their very heterogenous data have more generalisation problems, which federated aggregation is better able to counter. In the personalized FL frameworks, the FedPer and pFedMe techniques exhibited the best generalization stability, and the performance drop between cross-age studies was limited to a margin of less than 3% confirming that local adaptation layers are used to bridge the gap in the feature-space responses between cohorts of ages.

We evaluated fairness using q-FedAvg, which re-weights client contributions during aggregation based on performance degradation. Clients with worse performance receive higher weights to prevent well-performing clients from dominating the global model. When we increased in q from 0 (FedAvg) to 5, the accuracy variance across clients decreased from 4.1 to 2.1. This demonstrates that q-FedAvg improve the fairness between the clients.

Lastly, the bias reduction was studied within large datasets in terms of the error differences before and after implementing fairness-centred FL algorithms. Standard FedAvg was characterised by consistent error differences between children and adult clients, with sensitivity being the most notable (children: 81.3% vs. adults: 89.7%). When fairness constraints were incorporated this disparity was minimised to less than 3%, with q-FedAvg having the most consistent error distribution with demographic partitions. Comprehensively, the results reveal that fairness-conscious FL can be of great value in enhancing cross-population equity, minimising model bias, and improving generalisation with age-diverse datasets.

### 4.9 Ablation study

The ablation study Table 11 separates the impact of each significant design element to the overall performance of the federated architecture, specifically on the choice of the features, data balancing using SMOTE, the behaviour of batch normalisation in FedBN, and the optimization of the local training epochs. All reported accuracies correspond to the global federated model evaluated on a pooled held-out test set. Experiments were all performed on the same non-IID partitioning scheme. The findings are consistently showing that every design option has a quantifiable influence on enhancing accuracy, stability, and equity among clients.

**Table 11:** Ablation study results across federated learning methods. BN removal applies only to FedBN.

Ablation setting	FedAvg	FedProx	FedBN	FedPer	q-FedAvg
Full system (baseline)	88.57	89.57	90.50	91.00	90.67
Without feature selection	84.92	86.10	87.02	87.50	87.41
Without SMOTE	86.11	87.34	88.20	88.62	87.92
Without batch normalization	–	–	87.14	–	–
Local epochs = 1	85.40	85.02	86.10	86.72	86.31
Local epochs = 5	83.92	84.10	85.50	85.12	85.90

Turning off the ability to select features, using Mutual Information, and training models on all the raw features led to reduced accuracies in all federated learning methods. The heterogeneity of the clients was amplified by redundant and noisy attributes, which resulted in a FedAvg accuracy of 84.92%. MI-based feature selection retained only the most informative attributes, thus reducing the dimensionality by an estimated 35% and giving more reliable gradient changes per federated round. The latter improvement highlights the importance of our applied feature selection technique in federated systems with non-IID heterogeneous clients.

Elimination of SMOTE decreased the overall accuracy by 2%–3% points across all FL algorithms, with q-FedAvg decreasing to 87.92%. The impact of batch normalization in FedBN was investigated by removing BN layers. This resulted in a degradation of accuracy from 90.50% to 87.14%. This 3.36 pp drop demonstrates that BN layers are important to handle statistical heterogeneity across clients by maintaining client-specific normalization statistics while sharing global model parameters. Reducing local epochs to 1 resulted in accuracy drops across all methods (3-4 pp). With single epoch learning, clients failed to learn from their local data before aggregation, producing underfit updates before aggregation. Additionally, increasing local epochs to 5 caused greater degradation (4-5 pp). This occurs because excessive local training on heterogeneous distributions causes each cohort over-specializes on its own patterns, and when these updates are aggregated, they conflict and destabilize the global model. This shows that tuning the local number of epochs hyperparameter is essential for stable convergence in non-IID settings.

### 4.10 Computational efficiency analysis

**Figure 9:**
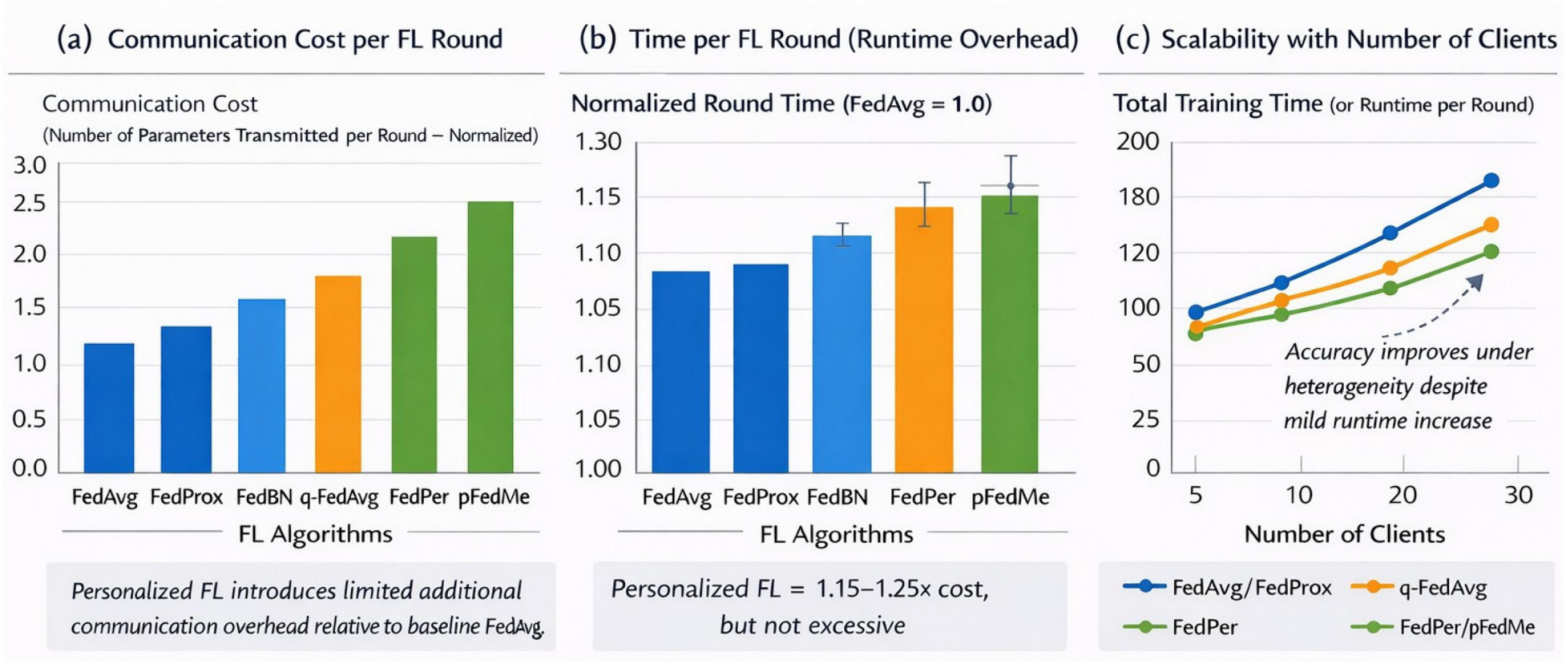
Computational efficiency and scalability analysis of federated learning methods. **(a)** Communication cost per FL round measured as the number of parameters transmitted per round (normalized) across different FL algorithms. **(b)** Time per FL round (runtime overhead) normalized to FedAvg = 1.0 for each FL algorithm. **(c)** Scalability with the number of clients showing total training time (or runtime per round) and improvement in accuracy under heterogeneity despite mild runtime increase.

The issue of computational efficiency is also vital in federated learning systems, especially in healthcare systems where various institutions with different resources are involved (40). We measured three efficiency aspects such as the cost of communication, the time per FL round, and the efficiency as the number of clients grows. The cost of communication Figure 9 was measured as the sum of the number of model parameters sent on each training round. Algorithms with personalization layers (e.g., FedPer and pFedMe) are slightly more costly to communicate than FedAvg due to the exchange of extra local head parameters; but still comparatively low in relation to the total network size. FedBN requires very little additional communication because only batch normalisation statistics are stored locally, as compared to q-FedAvg, which adds little additional cost in terms of reweighting of client losses during aggregation.

The time per round of the FL was measured on the hardware setup. In terms of the number of clients or local epochs, round time was minimised in FedAvg, which is simpler and has equal parameter sharing. FedProx was slower because of the proximal regularisation term in local optimization and the personalised approaches of FedPer and pFedMe were more slower because of the extra forwardbackward calculations in local heads. Although this increase was there, the personalised FL algorithms took each round 1.15–1.25x the time of FedAvg. FedBN exhibited a little more variance in runtime because of the batch normalisation operations against client-specific statistics especially where the client data sizes were not homogeneous.

Scalability analysis examined the behaviour of the model and the computation speed with an increment in clients involved to 30. FedAvg and FedProx had a linear dependence on communication and runtime, as well as constant convergence regardless of the number of clients. Individualised algorithms demonstrated slight increment in the overall training time with more customers as the cost of local adaption layers accumulated, but overall the global accuracy kept on improving particularly in heterogeneous and non-IID environment. It is notable that q-FedAvg was also fair and had low variance when using larger populations of clients and communication efficiency was enhanced with selective reweighting, which prioritised low-performing clients without overtransmitted parameters. These findings, in general, indicate that individualised FL approaches are only a little bit harder to run and communicate, but still can be deployed to many institutions and provide significant advantages in generalisation, fairness, and local customer performance.

### 4.11 Comparison with existing state-of-the-art

We place our work in the context of the literature by comparing it to centralised machine learning methods of ASD screening and recent federated learning (FL) models. This comparison Table 12 helps bring into focus how our framework contributes to the advancement of performance, privacy, and cross population generalisation in a manner that is not being done by the current methods.

**Table 12:** Comparison with recent literature on ASD machine learning and federated learning approaches.

Study (Year)	Modality & method	Data/setting	Key results	Limitations vs. our work
Rajagopalan et al. (41)	Medical/background features, XGBoost (centralized)	SPARK database (∼30 K)	AUC = 0.895; Sensitivity = 0.805; Specificity = 0.829	Centralized data; no privacy; no cross-site generalization
Leroy (42)	EHR + BiLSTM ensemble	Clinical notes labeled with DSM-5	Accuracy = 91%; Recall = 83%; Specificity = 100%	Requires centralized EHR; single-site; not Privacy-Aware
Liu et al. (43)	fMRI; multi-atlas deep ensemble	ABIDE I (multi-site)	75.2% (full); 96.4% (subset)	Imaging-only; high resource requirement; no FL or personalization
Shamseddine et al. (44)	Behavioral + facial data; federated learning	Simulated clients	∼70% (behavioral); ∼62% (facial)	Low accuracy; no personalization; simplified setting
FedHNN (45)	Questionnaire-based (fMRI + phenotype); hypergraph FL	Multi-site training	Better than local-only baselines; moderate accuracy	No personalization; medium convergence; limited fairness analysis
Eye-tracking FL (34)	Eye-tracking + kinematics; shallow DNN	3-client FL environment	Client accuracy up to ∼99% (controlled)	Small-scale; no personalization; constrained experimental setup
This work (Ours)	Behavioral questionnaire; Personalized FL (FedPer, pFedMe, q-FedAvg)	Children, adolescents, adults (non-IID clients)	Up to 97.2% (child), 89.5% (adolescent), 86.8% (adult); fairness + convergence studied	Privacy-Aware, cross-age, fairness-aware, personalization-enabled deployment

There are a number of recent studies that have utilised machine learning to detect ASD using behavioural, clinical, or questionnaire-based data. Indicatively, Rajagopalan et al. (41) have educated medical history and background characteristics on the SPARK cohort (more than 30,000 subjects) and constructed models (such as XGBoost) with an AUC of 0.895 with a sensitivity of 0.805 and specificity of 0.829. In yet another work, Temiz et al. (46) built a metagenomic biomarker-based ML classifier which employed explainable AI to make competitors predictions of ASD using gut microbiome data. On the deep learning side, Liu et al. (43). trained MADE on ASD: it is a multi atlas ensemble predicting fMRI data created with BiLSTM: their model was 75.2% accurate on the whole dataset and 96.4% accurate on a subset, however, with low generalisation to different datasets. Leroy (42) also created a transparent model based on deep learning: the BiLSTM ensemble model achieved 91% accuracy and 83% recall and 100% specificity on a subset, but There are models of different modalities (clinical, imaging, genetic), but none of them explicitly protect the privacy of data, either due to their centralised data aggregation, or the inability to deploy them across different sites.

Some studies have investigated the issue of ASD screening in decentralised training in the federated learning area, although these are not many. Other recent frameworks include a multi aspect FL scheme [Shamseddine et al. (44)] that prepares both behavioural and facial image data and reached a performance of only around 70% and 62% respectively under their conditions, but no personalization schemes were used (45). The other FL-based study combined eye-tracking and kinematic data in a safe and interpretable system, however it employed a simple DNN and reported very high client accuracies partially under highly controlled conditions (34). Our proposed framework has several key advantages over them: (1) we offer a systematic assessment and benchmarking of numerous personalised FL algorithms (FedPer, pFedMe, q FedAvg, etc.), (2) we deal with non-IID data across heterogeneous age groups (children, adolescents, adults), (3) we attain much higher accuracy (e.g., up to 97.2% when using children as a sample), and (4) we explicitly examine fairness, generalisation, communication cost, and convergence, which are important in deploying such systems in the The design of it thus not only further develops predictive performance but is also shown to be practically scalable, robust, and ethically mindful (e.g., privacy + fairness) in a manner the earlier FL ASD models have not.

Overall, our federated learning-based ASD screening system achieves better or complementary performance to existing state of the art methods in terms of high predictive accuracy, client personalization, as well as fairness analysis across non-IID cross-institutional conditions. Although previous studies have focused on either performance (centralised ML) or privacy (simple FL), ours is one of the first to optimise, evaluate and ASD-age diversity deploy advanced personalization-aware methods of FL systematically - bridging a critical divide between innovation in the methodology and clinical applicability.

## 5 Discussion

Our study demonstrates important findings about federated learning in the detection of Autism Spectrum Disorder (ASD) among heterogeneous populations. Another major problem with multi-institutional ASD screening is that data distributions are highly heterogeneous due to age-specific manifestations of behaviour, demographic diversity, and different assessment instruments. Our experiments indicate the inability of centralised machine learning models to perform satisfactorily when a non-IID distribution is presented in age cohorts, despite their performance on homogeneous datasets. Although the accuracy of Random Forest among children is 96.1%, among adults is only 81.0% because it is very hard to have one global model that is sensitive to age-related dynamics. These observations bring out the need to develop models that clearly consider heterogeneity and facilitate cross-age generalisation.

This study is different in a number of aspects of study design and evaluation as compared to the previous federated learning research on prediction tasks (ASD). First, the datasets are clearly split to capture the heterogeneity of age, which leads to a non-IID client structure more realistic to the screening situation in the real world. Second, we also compared a wide range of federated algorithms, such as personalization-based and fairness-aware federated strategies, to each other rather than comparing them to studies that make the assumption of a homogeneous client base or focus on a single federated strategy. Third, our evaluation standard highlights robustness with diverse clients with similar cross-validation and metrics of multiple performance, which allows us to have a more controlled comparison between centralised and federated strategy. These differences make it clear what we have to add to the existing literature and what value the proposed benchmarking framework would bring to it.

The benefits of personalized FL approaches, including FedPer, pFedMe, and q-FedAvg, are evident in the alleviation of the impact it has on non-IID data. These methods can be used to optimally customize the model parameters to fit the data of each client, though the models can be customized to the data distribution of each client as well, by adding client-specific optimization strategies or layers of local adaptation, without causing a loss in knowledge sharing between institutions. This personalization results in increased sensitivity on adult cohorts and increase in global accuracy, meaning that the model will be able to detect the subtle behavioural nuances that change with age. In addition, convergence analysis indicates that customised approaches ensure close-training dynamic despite the heterogeneous client data, indicating their strength in deploying them in practise where the sizes and distributions of clients cannot be forecasted.

The fairness analysis, especially q-FedAvg, shows the ability of the model to minimise the biasness between age groups and demographic subpopulations. Q-FedAvg is a weighted client loss aggregation mechanism that makes easy or challenging clients share equally with the rest of the global model updates. This allows this mechanism to decrease the performance gap between children, adolescents and adults, making the clinical reliability and trustworthiness more reliable. The improvements in the fairness measures observed are particularly relevant in the healthcare context, where biassed forecasting may result in a further increase in health disparities or incorrect risk assessment of a vulnerable demographic.

Lastly, there are significant clinical implications of our work. An extended, privacy-enabling, and high-performing FL-based system may enable various hospitals or research institutions to jointly devise effective ASD screening systems without leaking sensitive details about the patients. Fl models that are personalised can be implemented locally, whereby they change according to the specifics of the population whilst global knowledge is retained. This practice will lead to a decrease in false negatives, early interventions, and better accessibility to screening assistance in the regions. Also, computational efficiency analysis shows that these approaches are still feasible to deploy in a multi-client setting as it offers a balance between the cost of communication, run time, and predictive performance. Collectively, our results indicate that personalised federated learning can offer an effective route to the creation of clinically useful, privacy-conscious systems of ASD screening that can make generalisations across diverse groups.

### 5.1 Limitations and future work

Although the proposed federated ASD prediction framework demonstrates promising performance, several limitations should be considered when interpreting the results. First, scalability is evaluated using up to thirty simulated clients; however, the underlying data originate from only three real clinical cohorts (children, adolescents, and adults). As a result, system behaviour in larger and more heterogeneous institutional networks remains unvalidated with real multi-site data. While synthetic clients preserve certain statistical properties, they cannot fully reproduce the operational complexity, infrastructural heterogeneity, or data-governance disparities characteristic of real-world federated deployments.

A second limitation concerns privacy guarantees. The current implementation relies on standard federated learning without incorporating formal privacy-preserving mechanisms such as Differential Privacy (DP), Secure Aggregation, or cryptographic masking. Although raw data remain local to each cohort, vanilla FL does not inherently protect against threats such as gradient inversion, membership inference, or model-based information leakage. Future iterations should integrate DP or secure multi-party aggregation to provide quantifiable, end-to-end privacy assurances suitable for high-stakes clinical environments.

Another weakness arises from the use of synthetic oversampling techniques, such as SMOTE, to address class imbalance within individual client datasets. While these synthetic samples are generated locally and do not introduce direct privacy risks, they may introduce artefacts that are not fully representative of real patient characteristics, particularly in severely underrepresented groups. Moreover, oversampling in small or highly imbalanced subsets can lead to overfitting or diluted decision boundaries, potentially compromising model generalizability.

The paper also relies exclusively on questionnaire-based ASD screening instruments. Although widely adopted in clinical practice, such tools capture only a subset of behavioural and developmental traits relevant to ASD risk assessment and are inherently subjective. Their reliability may vary across cultures, languages, and socio-economic contexts. Consequently, the findings may not fully generalize to broader populations or to screening settings that incorporate more comprehensive clinical, physiological, or neurocognitive assessments.

Finally, despite the inclusion of fairness analyses demonstrating improved cross-cohort performance, residual population-level biases may persist. Differences in age distributions, symptom presentation, and class prevalence across cohorts can implicitly influence model behaviour, even under federated aggregation. Evaluating robustness using datasets that are more representative with respect to gender, ethnicity, geographic origin, and other sensitive attributes is therefore essential for real-world applicability.

Collectively, these limitations highlight several important directions for future research, including expansion to multi-institutional datasets, incorporation of formal privacy-preserving mechanisms, enrichment with additional clinical and questionnaire-based data, and more comprehensive fairness evaluations across diverse populations. Addressing these challenges will be critical for developing an ethically sound and fully privacy-preserving federated ASD prediction system suitable for clinical deployment.

The proposed framework also lends itself to multiple technical extensions. Enhancements such as secure aggregation, differential privacy mechanisms, and personalization strategies can be incorporated without fundamentally altering the federated learning architecture. In contrast, extensions involving multimodal data fusion, longitudinal monitoring, or real-world clinical deployment would require substantial redesign, access to richer clinical data, and tighter integration with healthcare workflows. Distinguishing between these development pathways provides a realistic and technically grounded road map for future research.

## 6 Conclusion

This paper has created a Privacy-Aware, federated learning-based, Autism Spectrum Disorder (ASD) detection framework acrross heterogeneous age groups. By comparing centralized machine learning models with personalized federated approaches such as FedPer, pFedMe, and q-FedAvg, we show that personalization improves accuracy, generalization, and fairness in multi-institutional settings. The framework achieves global accuracies of 97.2%, 89.5%, and 86.8% for children, adolescents, and adults, respectively, outperforming existing centralized and federated methods.

The proposed approach addresses challenges related to non-IID data, age-dependent behavioral differences, and privacy constraints, while aligning with regulations such as GDPR and HIPAA. Fairness-aware aggregation methods, including q-FedAvg, reduce performance gaps across demographic groups, supporting practical deployment in diverse clinical environments. Computational evaluations also indicate that these methods remain feasible in terms of runtime and communication overhead as the number of clients increases.

Several limitations should be acknowledged. First, the evaluation relies on public questionnaire-based datasets, which may not fully represent real clinical screening conditions. As a result, the findings may differ when applied to data collected in routine healthcare settings. Second, although the framework performs well in simulated non-IID scenarios, it has not been tested in an actual clinical deployment. Practical aspects such as clinician interaction, data governance, system integration, and longitudinal data use are not addressed. Third, while multiple federated models are compared, fairness and interpretability are assessed only at the performance level. Deeper analysis of subgroup fairness, feature attribution, and model explainability is needed for responsible use in healthcare. Finally, formal privacy guarantees are not yet incorporated, and privacy-enhancing techniques remain an important area for future validation.

Overall, this paper demonstrates that personalized federated learning provides a practical, ethical, and scalable solution for ASD screening, bridging the gap between high-performing machine learning and privacy-sensitive healthcare use. Future work will extend the framework to broader questionnaire integration, explore real hospital collaboration, and investigate adaptive personalization to further improve screening accuracy and inclusivity across populations.

## Data Availability

The datasets presented in this study can be found in online repositories. The names of the repository/repositories and accession number(s) can be found in the article/Supplementary Material.

## References

[1] Abualait T Alabbad M Kaleem I Imran H Khan H Kiyani MM, et al. Autism spectrum disorder in children: early signs and therapeutic interventions. Children. (2024) 11:1311. 10.3390/children11111311 39594885 PMC11592467

[2] Al-Qahtani A Al-Mahawes F Al-Qahtani F Berriche L Sainte SL-M. Cloud-based emotion recognition application for arabic autistic kids. In: *2025 8th International Conference on Data Science and Machine Learning Applications (CDMA)*. IEEE (2025). p. 174–9.

[3] Larcher FM Grözinger M. The spectrum effect: convergence of clinical and neuropsychological characteristics in adults referred for autism assessment. Res Autism Spectr Disord. (2025) 119:102502. 10.1016/j.rasd.2024.102502

[4] Daniel KS Jiang Q Wood MS. The increasing prevalence of autism spectrum disorder in the us and its implications for pediatric micronutrient status: a narrative review of case reports and series. Nutrients. (2025) 17:990. 10.3390/nu17060990 40290005 PMC11945165

[5] Vandewouw MM Brian J Crosbie J Schachar RJ Iaboni A Georgiades S, et al. Identifying replicable subgroups in neurodevelopmental conditions using resting-state functional magnetic resonance imaging data. JAMA Netw Open. (2023) 6:e232066. 10.1001/jamanetworkopen.2023.2066 36912839 PMC10011941

[6] Yang X Huang K Yang D Zhao W Zhou X. Biomedical big data technologies, applications, and challenges for precision medicine: a review. Glob Chall. (2024) 8:2300163. 10.1002/gch2.202300163 38223896 PMC10784210

[7] Abdelwahab A Al-Karawi KA Hasanin EM Semary HE. Autism spectrum disorder prediction in children using machine learning. J. Disabil. Res. (2024) 3(1):1–9. 10.57197/JDR-2023-0064

[8] Jacob SG Sulaiman MMBA Bennet B. Algorithmic approaches to classify autism spectrum disorders: a research perspective. Procedia Comput Sci. (2022) 201:470–7. 10.1016/j.procs.2022.03.061

[9] Ahammed MS Niu S Ahmed MR Dong J Gao X Chen Y. Bag-of-features model for asd fMRI classification using SVM. In: *2021 Asia-Pacific Conference on Communications Technology and Computer Science (ACCTCS)*. IEEE (2021). p. 52–7.

[10] Ahmed M Hussain S Ali F Gárate-Escamilla AK Amaya I Ochoa-Ruiz G, et al. Summarizing recent developments on autism spectrum disorder detection and classification through machine learning and deep learning techniques. Appl Sci. (2025) 15:8056. 10.3390/app15148056

[11] Ehsan K Sultan K Fatima A Sheraz M Chuah TC. Early detection of autism spectrum disorder through automated machine learning. Diagnostics. (2025) 15:1859. 10.3390/diagnostics15151859 40804824 PMC12346167

[12] Alzakari SA Allinjawi A Aldrees A Zamzami N Umer M Innab N, et al. Early detection of autism spectrum disorder using explainable AI and optimized teaching strategies. J Neurosci Methods. (2025) 413:110315. 10.1016/j.jneumeth.2024.110315 39532186

[13] Williamson SM Prybutok V. Balancing privacy and progress: a review of privacy challenges, systemic oversight, and patient perceptions in AI-driven healthcare. Appl Sci. (2024) 14:675. 10.3390/app14020675

[14] Javed H El-Sappagh S Abuhmed T. Robustness in deep learning models for medical diagnostics: security and adversarial challenges towards robust AI applications. Artif Intell Rev. (2025) 58:12. 10.1007/s10462-024-11005-9

[15] Sen A Heng S-H Tan S-C. A comprehensive review of cryptographic techniques in federated learning for secure data sharing and applications. IEEE Access. (2025) 13:135138–64. 10.1109/ACCESS.2025.3593953

[16] Abbas SR Abbas Z Zahir A Lee SW. Federated learning in smart healthcare: a comprehensive review on privacy, security, and predictive analytics with iot integration. In: Healthcare. Basel: MDPI AG (2024). Vol. 12. p. 2587. 10.3390/healthcare12242587 PMC11728217 39766014

[17] Ahmed IA Senan EM Rassem TH Ali MA Shatnawi HSA Alwazer SM, et al. Eye tracking-based diagnosis and early detection of autism spectrum disorder using machine learning and deep learning techniques. Electronics. (2022) 11:530. 10.3390/electronics11040530

[18] Alsharif N Al-Adhaileh MH Al-Yaari M Farhah N Khan ZI. Utilizing deep learning models in an intelligent eye-tracking system for autism spectrum disorder diagnosis. Front Med. (2024) 11:2024. 10.3389/fmed.2024.1436646 PMC11294196 39099594

[19] Tao X Sáenz-Lechón N Eckert M. Mapping the landscape of artificial intelligence for serious games in health: an enhanced meta review. Comput Hum Behav Rep. (2025) 18:100696. 10.1016/j.chbr.2025.100696

[20] Lakhan A Mohammed MA Abdulkareem KH Hamouda H Alyahya S. Autism spectrum disorder detection framework for children based on federated learning integrated CNN-LSTM. Comput Biol Med. (2023) 166:107539. 10.1016/j.compbiomed.2023.107539 37804778

[21] Jamil N Belkacem AN. Advancing real-time remote learning: A novel paradigm for cognitive enhancement using eeg and eye-tracking analytics. IEEE Access. (2024) 12:93116–32. 10.1109/ACCESS.2024.3422926

[22] Gupta S Bhuiyan M Chowa S Montaha S Rahman R Tanzir Mehedi S Rahman Z, Data from: Enhancing autism spectrum disorder classification with lightweight quantized CNNs and federated learning on ABIDE-1 dataset. Mathematics. (2024) 12(18):2886.

[23] Eden R Chukwudi I Bain C Barbieri S Callaway L de Jersey S, et al. A scoping review of the governance of federated learning in healthcare. npj Digit Med. (2025) 8:427. 10.1038/s41746-025-01836-3 40640574 PMC12246253

[24] Ashraf A Qingjie Z Bangyal WHK Iqbal M. Analysis of brain imaging data for the detection of early age autism spectrum disorder using transfer learning approaches for internet of things. IEEE Trans Consum Electron. (2024) 70:4478–89. 10.1109/TCE.2023.3328479

[25] Mohammadifar A Samadbin H Daliri A. Accurate autism spectrum disorder prediction using support vector classifier based on federated learning (SVCFL). *arXiv* [Preprint]. *arXiv:2311.04606* (2023).

[26] Karami M Karami A. Harmony in federated larning: a comprehensive review of techniques to tackle heterogeneity and non-iid data. Cluster Comput. (2025) 28:570. 10.1007/s10586-025-05250-y

[27] Daliri A Khalilian M Mohammadzadeh J Hosseini SS. Optimized active fuzzy deep federated learning for predicting autism spectrum disorder. Netw Model Anal Health Inform Bioinform. (2025) 14:31. 10.1007/s13721-025-00523-3

[28] Moussaoui J-E Kmiti M El Gholami K Maleh Y. A systematic review on hybrid ai models integrating machine learning and federated learning. J Cybersecur Priv. (2025) 5:41. 10.3390/jcp5030041

[29] Setu DM Islam T Rahman MM Dey SK Rahman T. Evaluating the efficacy and site-specific performance of machine learning approaches: a comprehensive review of autism detection models. Franklin Open. (2025) 11:100275. 10.1016/j.fraope.2025.100275

[30] Alshammari NK Alhusaini AA Pasha A Ahamed SS Gadekallu TR Abdullah-Al-Wadud M, et al. Explainable federated learning for enhanced privacy in autism prediction using deep learning. J Disabil Res. (2024) 3:20240081. 10.57197/JDR-2024-0081

[31] Syfullah MK Ali MS Hossain MM. Federated learning for autism spectrum disorder detection. In: *International Conference on Machine Intelligence and Emerging Technologies*. Springer (2024). p. 145–60.

[32] Kranthi M Venkatesan R Ramalakshmi K Wanika RAC. Privacy-preserving autism detection using federated CNN-LSTM networks: a spatiotemporal deep learning framework for decentralized behavioural diagnosis. In: *2025 International Conference on Sensors and Related Networks (SENNET) Special Focus on Digital Healthcare (64220)*. IEEE (2025). p. 1–6.

[33] Guan H Yap P-T Bozoki A Liu M. Federated learning for medical image analysis: A survey. Pattern Recognit. (2024) 151:110424. 10.1016/j.patcog.2024.110424 38559674 PMC10976951

[34] Almadhor A Alasiry A Alsubai S Al Hejaili A Kovac U Abbas S. Explainable and secure framework for autism prediction using multimodal eye tracking and kinematic data. Complex Intell Syst. (2025) 11:173. 10.1007/s40747-025-01790-3

[35] Gutierrez DMJ Solans D Heikkilä MA Vitaletti A Kourtellis N Anagnostopoulos A Non-IID data in federated learning: a taxonomy and analysis. *arXiv* [Preprint]. *arXiv:2411.12377* (2024).

[36] Zhao Y Li M Lai L Suda N Civin D Chandra V. Federated learning with non-IID data. *arXiv* [Preprint]. *arXiv:1806.00582* (2018).

[37] Thabtah F. Data from: Autistic spectrum disorder screening data for children (2017c). Available online at: https://archive.ics.uci.edu/dataset/419/autistic%26 (Accessed Febuary 23, 2025).

[38] Thabtah F. Data from: Autistic spectrum disorder screening data for adolescents (2017b). Available online at: https://archive.ics.uci.edu/dataset/420/autistic%2Bspectrum%2B (Accessed: Febuary 23, 2025).

[39] Thabtah F. Data from: Autism screening adult data set (2017a). Available online at: https://archive.ics.uci.edu/ml/datasets/autism%2Bscreening%2Badult (Accessed Febuary 23, 2025).

[40] Rekik S Alsulaiman N Albadrani N. A health record management system using blockchain and smart contract. In: *2024 Seventh International Women in Data Science Conference at Prince Sultan University (WiDS PSU)*. (2024). p. 204–8.

[41] Rajagopalan SS Zhang Y Yahia A Tammimies K. Machine learning prediction of autism spectrum disorder from a minimal set of medical and background information. JAMA Netw Open. (2024) 7:e2429229. 10.1001/jamanetworkopen.2024.29229 39158907 PMC11333987

[42] Leroy G Andrews JG KeAlohi-Preece M Jaswani A Song H Galindo MK, et al. Transparent deep learning to identify autism spectrum disorders (ASD) in EHR using clinical notes. J Am Med Inform Assoc. (2024) 31:1313–21. 10.1093/jamia/ocae080 38626184 PMC11105145

[43] Liu X Hasan MR Gedeon T Hossain MZ. Made-for-ASD: a multi-atlas deep ensemble network for diagnosing autism spectrum disorder. Comput Biol Med. (2024) 182:109083. 10.1016/j.compbiomed.2024.109083 39232404

[44] Shamseddine H Otoum S Mourad A. A federated learning scheme for neuro-developmental disorders: multi-aspect asd detection. *arXiv* [Preprint]. *arXiv:2211.00643* (2022).

[45] Wang H Jing H Yang J Liu C Hu L Tao G, et al. Identifying autism spectrum disorder from multi-modal data with privacy-preserving. npj Ment Health Res. (2024) 3:15. 10.1038/s44184-023-00050-x 38698164 PMC11066078

[46] Temiz M Bakir-Gungor B Ersoz NS Yousef M. Machine learning-based prediction of autism spectrum disorder and discovery of related metagenomic biomarkers with explainable AI. Appl Sci. (2025) 15:9214. 10.3390/app15169214

[47] Rehman IU Sobnath D Nasralla MM Winnett M Anwar A Asif W Features of mobile apps for people with autism in a post COVID-19 scenario: Current status and recommendations for apps using AI. Diagnostics. (2021) 11(10):1923. 10.3390/diagnostics11101923 34679621 PMC8535154

